# Association of Parity with Type 2 Diabetes Mellitus in Japan

**DOI:** 10.1007/s43032-024-01752-z

**Published:** 2024-12-11

**Authors:** Hongxin Wang, Noriyuki Iwama, Keiichi Yuwaki, You Nakamichi, Hirotaka Hamada, Hasumi Tomita, Kazuma Tagami, Rie Kudo, Natsumi Kumagai, Hirohito Metoki, Naoki Nakaya, Atsushi Hozawa, Shinichi Kuriyama, Nobuo Yaegashi, Masatoshi Saito

**Affiliations:** 1 Department of Obstetrics and Gynecology, Tohoku University Graduate School of Medicine, Tohoku University Hospital, 1-1, Seiryomachi, Sendai, Miyagi Japan; 2 Women’s Health Care Medical Science, Tohoku University Graduate School of Medicine, 1-1, Seiryomachi, Sendai, Miyagi Japan; 3 Tohoku Medical Megabank Organization, Tohoku University, 2-1, Seiryomachi, Sendai, Miyagi Japan; 4 Underwriting and Medical Department, The Dai-Ichi Life Insurance Company, Limited, 3-2-3, Toyosu, Koto-Ku, Tokyo, Japan; 5 Division of Public Health, Hygiene and Epidemiology, Tohoku Medical Pharmaceutical University, 1-15-1, Fukumuro, Sendai, Miyagi Japan; 6 Division of Molecular Epidemiology, Tohoku University Graduate School of Medicine, 1-1, Seiryomachi, Sendai, Miyagi Japan; 7 International Research Institute of Disaster Science, Tohoku University, 468-1, Aramaki, Sendai, Miyagi Japan; 8 Environment and Genome Research Center, Tohoku University Graduate School of Medicine, 2-1, Seiryomachi, Sendai, Miyagi Japan; 9 Department of Maternal and Fetal Therapeutics, Tohoku University Graduate School of Medicine, 1-1, Seiryomachi, Sendai, Miyagi Japan

**Keywords:** Parity, Diabetes mellitus, Gestational diabetes mellitus, Cohort study

## Abstract

This study investigates the association between parity and type 2 diabetes mellitus (T2DM) in Japanese women, considering the clinical history of gestational diabetes mellitus (GDM) and menopausal status, which are known risk factors for T2DM. Overall, 30,116 Japanese women (6,588 premenopausal and 23,528 postmenopausal) were included in this cross-sectional study. They were divided into two groups according to menopausal status (premenopausal and postmenopausal women), and the association between parity and T2DM was evaluated using a multiple logistic regression model with possible confounders, including a clinical history of GDM. The association between parity and T2DM was not statistically significant in premenopausal women. In contrast, a linear graded association between parity and T2DM was found in postmenopausal women. Furthermore, the association between parity and T2DM in postmenopausal women was attenuated after adjusting for body weight gain after the age of 20 years. A clinical history of GDM was significantly associated with a high risk for T2DM, regardless of adjustment for body weight gain after the age of 20 years in both premenopausal and postmenopausal women. Parity is associated with an increased risk of T2DM in postmenopausal women but not in premenopausal women. Maintaining appropriate body weight would be beneficial in attenuating the risk of T2DM in postmenopausal women. A clinical history of GDM is a risk factor for T2DM in both pre- and postmenopausal women; therefore, women with a clinical history of GDM require continuous medical care to survey for T2DM.

**Supplementary Information:**

The online version contains supplementary material available at 10.1007/s43032-024-01752-z.

## Introduction

Diabetes mellitus (DM), one of the most common chronic diseases worldwide, is associated with an increased risk of death, especially from cardiovascular diseases [1, 2]. DM is also included in the risk prediction model for cardiovascular disease and is recognized as a significant independent risk factor for all subtypes of ischemic strokes in middle-aged Japanese individuals [3, 4]. Although type 2 DM (T2DM) is more prevalent in men than in women [5], women with T2DM have a greater risk of incident coronary heart disease [6], acute myocardial infarction [7], and stroke than men [8]. Therefore, prevention of T2DM is important from a women's healthcare perspective. Childbearing is a life event that is unique to women. Several studies have reported an association between the number of childbearing (i.e., parity) and T2DM. However, whether the number of parities increases the risk of T2DM remains inconclusive. Prior studies have corroborated the finding of a graded association between parity, particularly grand multiparity, and T2DM [9–12]. In contrast, other studies have reported no relationship between parity and an increased risk of T2DM [13–15].

Asian Americans are known to have a significantly higher risk for T2DM despite having a substantially lower body mass index (BMI) than their Caucasian counterparts [16]. Compared to Western women, Japanese women have a significantly lower BMI and different lifestyle, suggesting that the association between parity and T2DM risk may differ between the Japanese and Western populations [17, 18]. However, only one study on the association between parity and T2DM has been previously conducted in Japan [17]. Furthermore, no previous studies have investigated the association between parity and T2DM, while considering the clinical history of gestational diabetes mellitus (GDM), a risk factor for T2DM [9]. This study aims to examine the association between parity and T2DM in Japan.

## Materials and Methods

### Study Design and Participants

This is a cross-sectional study that is based on data from a type 1 survey of the Tohoku Medical Megabank Community-Based Cohort Study (TMM CommCohort Study). The TMM CommCohort Study is an ongoing prospective cohort study conducted since 2013 in the Miyagi and Iwate Prefectures, Japan. It was launched to achieve creative reconstruction and solve medical problems in the aftermath of the Great East Japan Earthquake (GEJE) and the resulting tsunami that occurred on March 11, 2011, causing devastating damage to the Pacific coast of the Tohoku region. Details of the study design have been previously published [19–21]. Both men and women were recruited in the TMM CommCohort study, and only women were included in this study. Women participants who fulfilled the following criteria were included in this study: (1) those who were aged ≥ 20 years and < 75 years and were living in Miyagi Prefecture and Iwate Prefecture between May 2013 and March 2016 when the Tohoku Medical Megabank Community-based Cohort Study Baseline survey was conducted; and (2) those who agreed to join the Tohoku Medical Megabank Community-based Cohort Study during the municipal health checkup. Written informed consent was obtained from all the participants. This study was approved by the Institutional Review Board of Tohoku University School of Medicine (approval numbers:2021–1–608, 2022–1–069, 2022–1–216, and 2022–1–825).

### Inclusion and Exclusion of Study Participants

**Fig. 1:**
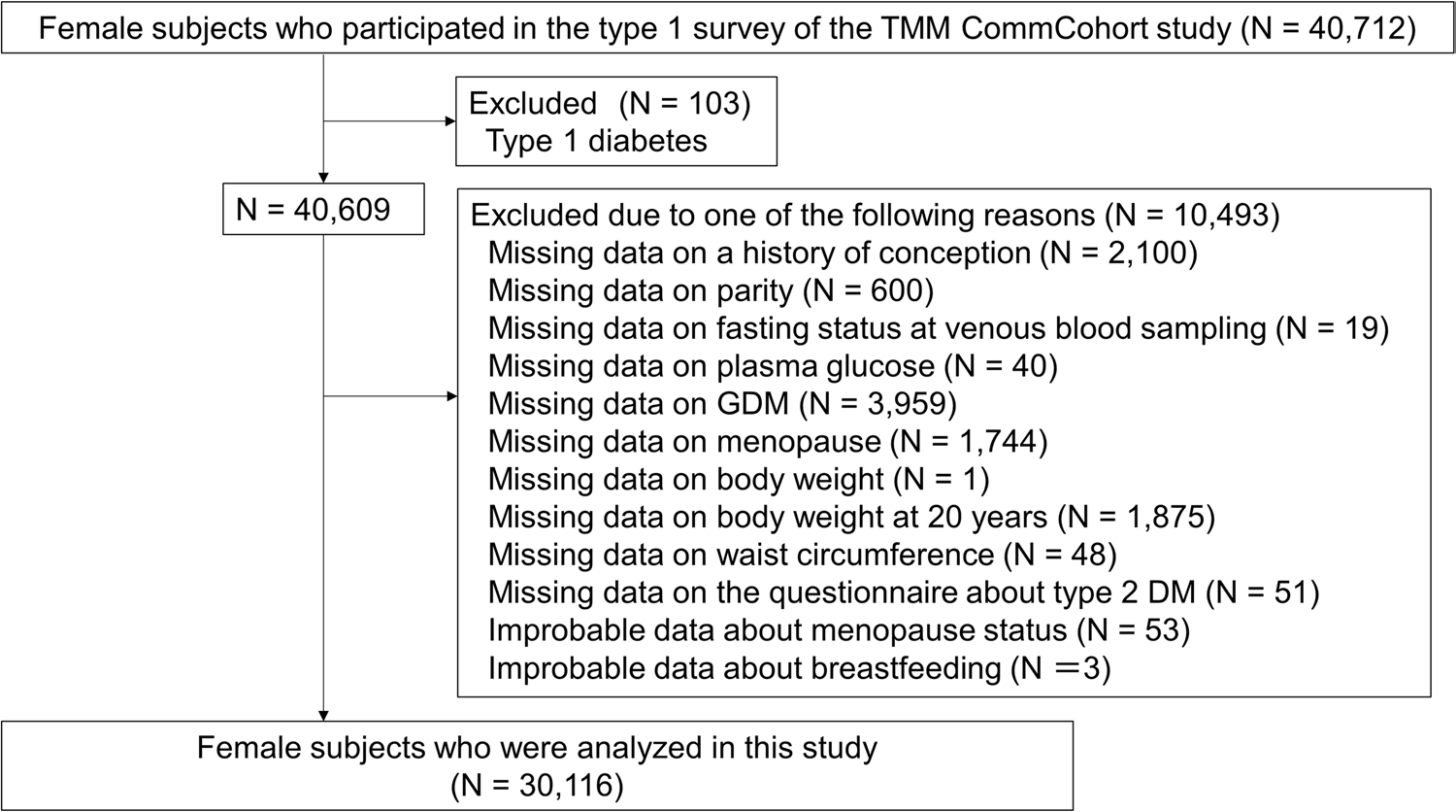
Flow chart of this study

Figure 1 shows the flowchart of this study. A total of 40,712 women participated in the Type 1 survey of the TMM CommCohort Study. We excluded women if they were diagnosed with type 1 diabetes (*N* = 103), had missing data on history of conception (*N* = 2,100), had missing data on parity (*N* = 600), had missing data on fasting status at venous blood sampling (*N* = 19), had missing data on plasma glucose (*N* = 40), had missing data on a clinical history of GDM (*N* = 3,959), had missing data on menopause (*N* = 1,744), had missing data on current body weight (*N* = 1), had missing data on body weight at 20 years (*N* = 1875), had missing data on current waist circumference (WC) (*N* = 48), had missing data on the questionnaire concerning T2DM (*N* = 51), had improbable data on menopausal status (*N* = 53), and had improbable data about breastfeeding (*N* = 3). Finally, 30,116 women (6,588 premenopausal women and 23,528 postmenopausal women) were analyzed in this study. The differences in characteristics between women who were analyzed and those who were excluded owing to missing or clinically improbable data are shown in Supplementary Table S1.

### Data Collection

#### Participants

A total of 40,712 women were included in this study. To account for the impact of menopause on metabolic health [22, 23] and the cessation of future childbearing potential in postmenopausal women, women were divided into two groups: premenopausal women and postmenopausal women in our study.

#### Parity

Parity is an exposure of interest in this study. Information on the number of children was obtained from self-reported questionnaires. In this study, we defined the number of children as parity. Parity was classified as nulliparous (i.e., parity = 0), one, two, three, and ≥ four, respectively. Neither the number of stillbirths nor the number of multiple pregnancies were collected in this study.

### Operational Definitions

#### T2DM

The outcome of this study is T2DM. The participants’ medical information was collected using self-reported questionnaires. Venous blood samples were collected from the municipal health checkup venues. Information on whether venous blood was drawn in the fasting state was collected. In this study, participants were diagnosed with T2DM if any of the following conditions were met: (1) participants who answered ‘yes’ in the self-reported questionnaire for having T2DM, (2) participants who answered ‘under treatment of DM’ in the self-reported questionnaire on lifestyle diseases, (3) plasma glucose (PG) level ≥ 126 mg/dl if venous blood was drawn in the fasting status, (4) PG level ≥ 200 mg/dl if venous blood was drawn in the non-fasting status, and (5) glycosylated hemoglobin (HbA1c) (the National Glycohemoglobin Standardization Program: NGSP) level ≥ 6.5%. PG and HbA1c levels were measured using enzymatic methods [20]. As the outcome was T2DM, participants with type1 DM were excluded from this study.

#### Clinical History of GDM

Information on the clinical history of GDM was obtained using a self-report questionnaire. GDM was diagnosed according to the Japan Society of Obstetrics and Gynecology criteria of 1984, if two or more of the following values during a 75-g oral glucose tolerance test (OGTT) were met: fasting PG level ≥ 100 mg/dl, 1-h PG level ≥ 180 mg/dl, and 2-h PG level ≥ 150 mg/dl, regardless of gestational age [24].

#### Premenopausal and Postmenopausal Women

Both premenopausal and postmenopausal women were identified using a self-recorded questionnaire. Participants answered about current menstrual status by choosing from the three options: "I have menstruation,” "Menstruation is disappearing,” and "No menstruation for more than a year.” In this study, premenopausal women were defined as those who answered "I have menstruation" or "Menstruation is disappearing." Postmenopausal women were defined as those who answered "no menstruation for more than a year”.

Supporting information: Details of collection methods for other variables are described in the Supporting Information.

### Statistical Analysis

After the women were divided into two groups (premenopausal and postmenopausal women), we performed all statistical analyses in each group separately.

The linear association between parity and T2DM was tested using the Cochran-Armitage test. We used a multiple logistic regression model to investigate the association between parity and T2DM. Nulliparous women (parity = 0) were set as the reference category. Model 1 was adjusted for age. Model 2 was adjusted for the following covariates: height, physical activity, marital status (married or widowed, unmarried, and divorced), smoking status, alcohol consumption, own birth weight, highest educational level, family history of T2DM, family history of hypertension, breastfeeding experience, use of oral contraceptives, use of hormone replacement therapy, thyroid dysfunction [25], endometriosis, mental disease, menstrual cycle, age at menarche (< 15 years or ≥ 15 years), age at last delivery (< 35 years or ≥ 35 years), sleeping time, nap time, year of study participation, prefecture (Miyagi or Iwate), and number of relocations after the GEJE, in addition to Model 1. When postmenopausal women were analyzed, menopausal age (age at menopause < 40 years or ≥ 40 years) was further included in Model 2 with reference to a previous study [26]. Model 3 was adjusted for clinical history of GDM in addition to Model 2. Model 4 was adjusted for BMI at 20 years of age, as per the 1-SD increase, in addition to Model 3. With reference to a previous study, Model 5 was adjusted for weight gain from the age of 20 years, as per 1 kg increase, in addition to model 4 [27]. No strong multicollinearity was observed. The linear association between parity and T2DM was tested in each model.

Considering the missing data on covariates, we applied multiple imputation using a Markov chain Monte Carlo simulation. After 20 datasets were created by multiple imputations, each dataset was analyzed. Finally, 20 results were combined and are described in the manuscript. The details of the additional analyses are described in the Supporting Information.

Statistical analysis was performed using SAS software version 9.4 (SAS Institute Inc., Cary, North Carolina, USA) and R version 4.1.1 [28].

## Results

### Characteristics of the Premenopausal Women by Parity

The characteristics of premenopausal women and those according to parity are shown in Table 1. The study was conducted in Japan. No definite trend was observed between increased parity and T2DM prevalence. Age and hypertension were more prevalent as parity increased. Furthermore, a higher proportion of women with breastfeeding experience, who used menopausal medications (such as hormonal drugs), with a history of hypertensive disorders of pregnancy (HDP), and that are residents of Iwate Prefecture were observed as parity increased. The proportion of those that are currently obese was higher in women with a parity ≥ four, compared to that in women with lower parity. As parity increased, the proportion of patients with a family history of T2DM decreased. The proportions of unmarried and divorced women were higher in nulliparous women and in women with parity of one, respectively. As parity decreased, the proportion of patients with a higher level of education increased.

**Table 1:** Characteristics of premenopausal women by parity

Variables	All (*N* = 6,588)	Parity				
		0 (*N* = 1,585)	1 (*N* = 1,102)	2 (*N* = 2,452)	3 (*N* = 1,185)	≥ 4 (*N* = 264)
Age, years	41.3 (7.4)	38.1 (8.3)	39.9 (6.9)	42.4 (6.6)	43.8 (6.7)	44.3 (6.7)
Category of age, *N* (%)						
20–29.9 years	419 (6.4)	292 (18.4)	70 (6.4)	47 (1.9)	8 (0.7)	2 (0.8)
30–39.9 years	2,238 (34.0)	553 (34.9)	445 (40.4)	811 (33.1)	355 (30.0)	74 (28.0)
40–49.9 years	2,938 (44.6)	617 (38.9)	485 (44.0)	1,174 (47.9)	549 (46.3)	113 (42.8)
50–59.9 years	993 (15.1)	123 (7.8)	102 (9.3)	420 (17.1)	273 (23.0)	75 (28.4)
Waist circumference, cm	78.4 (9.8)	77.9 (11.1)	78.5 (9.9)	78.3 (9.0)	78.9 (9.1)	80.4 (10.2)
Waist circumference ≥ 90 cm, *N* (%)	782 (11.9)	215 (13.6)	129 (11.7)	252 (10.3)	140 (11.8)	46 (17.4)
Height, cm	157.2 (5.3)	157.4 (5.4)	157.4 (5.4)	157.1 (5.2)	157.2 (5.2)	156.6 (4.8)
Current body weight, kg	55.2 (10.2)	55.7 (12.0)	55.0 (10.3)	54.6 (9.4)	55.5 (9.4)	56.7 (9.8)
Current BMI, kg/m^2^	22.3 (4.0)	22.5 (4.6)	22.2 (4.0)	22.1 (3.7)	22.4 (3.6)	23.1 (4.0)
Category of current BMI, *N* (%)						
Underweight (< 18.5 kg/m^2^)	837 (12.7)	258 (16.3)	152 (13.8)	297 (12.1)	108 (9.1)	22 (8.3)
Normal range (18.5–24.9 kg/m^2^)	4,406 (66.9)	969 (61.1)	737 (66.9)	1,717 (70.0)	818 (69.0)	165 (62.5)
Obese ≥ 25.0 kg/m^2^)	1,345 (20.4)	358 (22.6)	213 (19.3)	438 (17.9)	259 (21.9)	77 (29.2)
Body weight at 20 years, kg	52.0 (8.0)	53.3 (9.6)	51.9 (8.2)	51.2 (6.9)	51.7 (7.3)	52.5 (7.3)
BMI at 20 years, kg/m^2^	21.0 (3.0)	21.5 (3.6)	20.9 (3.2)	20.7 (2.6)	20.9 (2.7)	21.4 (3.0)
Category of BMI at 20 years, *N* (%)						
Underweight (< 18.5 kg/m^2^)	1,054 (16.0)	265 (16.7)	189 (17.2)	403 (16.4)	165 (13.9)	32 (12.1)
Normal range (18.5–24.9 kg/m^2^)	5,008 (76.0)	1,114 (70.3)	823 (74.7)	1,911 (77.9)	954 (80.5)	206 (78.0)
Obese (≥ 25.0 kg/m^2^)	526 (8.0)	206 (13.0)	90 (8.2)	138 (5.6)	66 (5.6)	26 (9.8)
Body weight gain after 20 years, kg	3.2 (8.1)	2.4 (8.3)	3.1 (8.5)	3.4 (7.6)	3.8 (8.2)	4.2 (8.2)
Physical activity level, METS, Median (IQR)	26.8 (20.9–34.3)	26.8 (21.9–34.2)	26.0 (20.4–33.9)	26.6 (20.8–33.8)	27.2 (20.8–36.3)	27.9 (21.2–36.9)
Smoking status, *N* (%)						
Never smoker	4,491 (68.2)	1,133 (71.5)	697 (63.2)	1,666 (67.9)	822 (69.4)	173 (65.5)
Ever smoker	1,152 (17.5)	215 (13.6)	227 (20.6)	465 (19.0)	197 (16.6)	48 (18.2)
Current smoker	925 (14.0)	233 (14.7)	178 (16.2)	311 (12.7)	160 (13.5)	43 (16.3)
Missing	20 (0.3)	4 (0.3)	0 (0.0)	10 (0.4)	6 (0.5)	0 (0.0)
Alcohol consumption, *N* (%)						
Never drinker	3,285 (49.9)	825 (52.1)	557 (50.5)	1,206 (49.2)	565 (47.7)	132 (50.0)
Ever drinking	184 (2.8)	34 (2.1)	77 (7.0)	53 (2.2)	17 (1.4)	3 (1.1)
Current drinking	3,097 (47.0)	719 (45.4)	464 (42.1)	1,187 (48.4)	599 (50.5)	128 (48.5)
Missing	22 (0.3)	7 (0.4)	4 (0.4)	6 (0.2)	4 (0.3)	1 (0.4)
T2DM prevalence, *N* (%)	140 (2.1)	36 (2.3)	21 (1.9)	39 (1.6)	37 (3.1)	7 (2.7)
Hypertension prevalence, *N* (%)	800 (12.1)	162 (10.2)	116 (10.5)	294 (12.0)	182 (15.4)	46 (17.4)
Own birth weight, *N* (%)						
< 2,500 g	565 (8.6)	173 (10.9)	100 (9.1)	203 (8.3)	68 (5.7)	21 (8.0)
2,500–3,499 g	4,737 (71.9)	1,092 (68.9)	792 (71.9)	1,786 (72.8)	871 (73.5)	196 (74.2)
≥ 3,500 g	738 (11.2)	198 (12.5)	134 (12.2)	270 (11.0)	114 (9.6)	22 (8.3)
Unknown	435 (6.6)	82 (5.2)	65 (5.9)	161 (6.6)	108 (9.1)	19 (7.2)
Missing	113 (1.7)	40 (2.5)	11 (1.0)	32 (1.3)	24 (2.0)	6 (2.3)
History of thyroid dysfunction, *N* (%)						
Yes	204 (3.1)	44 (2.8)	30 (2.7)	84 (3.4)	37 (3.1)	9 (3.4)
No	6,203 (94.2)	1,370 (86.4)	1,069 (97.0)	2,363 (96.4)	1,146 (96.7)	255 (96.6)
Missing	181 (2.7)	171 (10.8)	3 (0.3)	5 (0.2)	2 (0.2)	0 (0.0)
History of endometriosis, *N* (%)						
Yes	324 (4.9)	85 (5.4)	73 (6.6)	118 (4.8)	40 (3.4)	8 (3.0)
No	6,089 (92.4)	1,328 (83.8)	1,028 (93.3)	2,332 (95.1)	1,145 (96.6)	256 (97.0)
Missing	175 (2.7)	172 (10.9)	1 (0.1)	2 (0.1)	0 (0.0)	0 (0.0)
History of mental diseases, *N* (%)						
Yes	392 (6.0)	161 (10.2)	68 (6.2)	116 (4.7)	29 (2.4)	18 (6.8)
No	6,019 (91.4)	1,257 (79.3)	1,031 (93.6)	2,331 (95.1)	1,154 (97.4)	246 (93.2)
Missing	177 (2.7)	167 (10.5)	3 (0.3)	5 (0.2)	2 (0.2)	0 (0.0)
Breastfeeding experience, *N* (%)						
Yes	4,747 (72.1)	0 (0.0)	1,011 (91.7)	2,346 (95.7)	1,132 (95.5)	258 (97.7)
No	1,762 (26.7)	1,517 (95.7)	85 (7.7)	104 (4.2)	50 (4.2)	6 (2.3)
Missing	79 (1.2)	68 (4.3)	6 (0.5)	2 (0.1)	3 (0.3)	0 (0.0)
Use of oral contraceptives, *N* (%)						
Yes	287 (4.4)	59 (3.7)	58 (5.3)	97 (4.0)	54 (4.6)	19 (7.2)
No	6,153 (93.4)	1,425 (89.9)	1,031 (93.6)	2,332 (95.1)	1,124 (94.9)	241 (91.3)
Missing	148 (2.2)	101 (6.4)	13 (1.2)	23 (0.9)	7 (0.6)	4 (1.5)
Use of hormone replacement therapy, *N* (%)						
Yes	165 (2.5)	26 (1.6)	19 (1.7)	65 (2.7)	42 (3.5)	13 (4.9)
No	6,252 (94.9)	1,452 (91.6)	1,065 (96.6)	2,354 (96.0)	1,135 (95.8)	246 (93.2)
Missing	171 (2.6)	107 (6.8)	18 (1.6)	33 (1.3)	8 (0.7)	5 (1.9)
≥ 15 years at menarche, *N* (%)						
< 15 years	6,144 (93.3)	1,456 (91.9)	1,016 (92.2)	2,325 (94.8)	1,103 (93.1)	244 (92.4)
≥ 15 years	404 (6.1)	116 (7.3)	76 (6.9)	120 (4.9)	76 (6.4)	16 (6.1)
Missing	40 (0.6)	13 (0.8)	10 (0.9)	7 (0.3)	6 (0.5)	4 (1.5)
≥ 35 years at last delivery, *N* (%)						
< 35 years	3,720 (56.5)	0 (0.0)	796 (72.2)	1,918 (78.2)	863 (72.8)	143 (54.2)
≥ 35 years	1,172 (17.8)	0 (0.0)	269 (24.4)	479 (19.5)	304 (25.7)	120 (45.5)
Missing	1,696 (25.7)	1,585 (100.0)	37 (3.4)	55 (2.2)	18 (1.5)	1 (0.4)
Menstrual cycle, *N* (%)						
Regular	5,141 (78.0)	1,211 (76.4)	837 (76.0)	1,970 (80.3)	925 (78.1)	198 (75.0)
Irregular	1,393 (21.1)	364 (23.0)	254 (23.0)	462 (18.8)	248 (20.9)	65 (24.6)
Missing	54 (0.8)	10 (0.6)	11 (1.0)	20 (0.8)	12 (1.0)	1 (0.4)
History of GDM, *N* (%)	54 (0.8)	0 (0.0)	14 (1.3)	20 (0.8)	17 (1.4)	3 (1.1)
History of HDP, *N* (%)						
Yes	290 (4.4)	0 (0.0)	48 (4.4)	137 (5.6)	80 (6.8)	25 (9.5)
No	6,295 (95.6)	1,585 (100.0)	1,053 (95.6)	2,313 (94.3)	1,105 (93.2)	239 (90.5)
Missing	3 (0.0)	0 (0.0)	1 (0.1)	2 (0.1)	0 (0.0)	0 (0.0)
Family history of T2DM, *N* (%)						
Yes	901 (13.7)	253 (16.0)	151 (13.7)	319 (13.0)	143 (12.1)	35 (13.3)
No	5,520 (83.8)	1,176 (74.2)	947 (85.9)	2,128 (86.8)	1,040 (87.8)	229 (86.7)
Missing	167 (2.5)	156 (9.8)	4 (0.4)	5 (0.2)	2 (0.2)	0 (0.0)
Family history of hypertension, *N* (%)						
Yes	2,923 (44.4)	746 (47.1)	468 (42.5)	1,076 (43.9)	512 (43.2)	121 (45.8)
No	3,558 (54.0)	740 (46.7)	632 (57.4)	1,372 (56.0)	671 (56.6)	143 (54.2)
Missing	107 (1.6)	99 (6.2)	2 (0.2)	4 (0.2)	2 (0.2)	0 (0.0)
Marital status, *N* (%)						
Married	5,015 (76.1)	501 (31.6)	936 (84.9)	2,240 (91.4)	1,099 (92.7)	239 (90.5)
Unmarried	1,042 (15.8)	1,005 (63.4)	23 (2.1)	8 (0.3)	4 (0.3)	2 (0.8)
Divorced	396 (6.0)	60 (3.8)	126 (11.4)	149 (6.1)	45 (3.8)	16 (6.1)
Widowed	120 (1.8)	10 (0.6)	15 (1.4)	55 (2.2)	33 (2.8)	7 (2.7)
Missing	15 (0.2)	9 (0.6)	2 (0.2)	0 (0.0)	4 (0.3)	0 (0.0)
Highest level of education, *N* (%)						
Low	210 (3.2)	45 (2.8)	36 (3.3)	67 (2.7)	46 (3.9)	16 (6.1)
Medium	4,811 (73.0)	1,091 (68.8)	785 (71.2)	1,815 (74.0)	909 (76.7)	211 (79.9)
High	1,524 (23.1)	434 (27.4)	277 (25.1)	554 (22.6)	222 (18.7)	37 (14.0)
Missing	43 (0.7)	15 (0.9)	4 (0.4)	16 (0.7)	8 (0.7)	0 (0.0)
Breakfast skipping, *N* (%)						
Not breakfast skipping	5,261 (79.9)	1,058 (66.8)	912 (82.8)	2,092 (85.3)	1,000 (84.4)	199 (75.4)
Breakfast skipping	1,303 (19.8)	525 (33.1)	187 (17.0)	355 (14.5)	175 (14.8)	61 (23.1)
Missing	24 (0.4)	2 (0.1)	3 (0.3)	5 (0.2)	10 (0.8)	4 (1.5)
Average sleeping time/day, *N* (%)						
< 7 h	4,986 (75.7)	1,108 (69.9)	798 (72.4)	1,904 (77.7)	958 (80.8)	218 (82.6)
≥ 7 and < 8 h	1,201 (18.2)	327 (20.6)	226 (20.5)	440 (17.9)	178 (15.0)	30 (11.4)
≥ 8 h	395 (6.0)	148 (9.3)	77 (7.0)	107 (4.4)	47 (4.0)	16 (6.1)
Missing	6 (0.1)	2 (0.1)	1 (0.1)	1 (0.0)	2 (0.2)	0 (0.0)
Nap time, *N* (%)						
Not taking a nap	4,484 (68.1)	1,086 (68.5)	748 (67.9)	1,686 (68.8)	805 (67.9)	159 (60.2)
Nap time is < one hour/day	1,423 (21.6)	310 (19.6)	216 (19.6)	543 (22.1)	280 (23.6)	74 (28.0)
Nap time is ≥ one hour/day	655 (9.9)	184 (11.6)	133 (12.1)	212 (8.6)	95 (8.0)	31 (11.7)
Missing	26 (0.4)	5 (0.3)	5 (0.5)	11 (0.4)	5 (0.4)	0 (0.0)
Number of relocations after the GEJE, *N* (%)						
0	4,621 (70.1)	1,078 (68.0)	684 (62.1)	1,772 (72.3)	890 (75.1)	197 (74.6)
1	865 (13.1)	223 (14.1)	203 (18.4)	300 (12.2)	112 (9.5)	27 (10.2)
2	507 (7.7)	139 (8.8)	99 (9.0)	171 (7.0)	84 (7.1)	14 (5.3)
3	307 (4.7)	71 (4.5)	58 (5.3)	109 (4.4)	56 (4.7)	13 (4.9)
≥ 4	207 (3.1)	54 (3.4)	42 (3.8)	71 (2.9)	33 (2.8)	7 (2.7)
Missing	81 (1.2)	20 (1.3)	16 (1.5)	29 (1.2)	10 (0.8)	6 (2.3)
Year, *N* (%)						
2013	1,048 (15.9)	356 (22.5)	147 (13.3)	320 (13.1)	182 (15.4)	43 (16.3)
2014	2,773 (42.1)	653 (41.2)	490 (44.5)	1,020 (41.6)	490 (41.4)	120 (45.5)
2015	2,767 (42.0)	576 (36.3)	465 (42.2)	1,112 (45.4)	513 (43.3)	101 (38.3)
Prefecture, *N* (%)						
Miyagi	4,132 (62.7)	1,051 (66.3)	735 (66.7)	1,542 (62.9)	674 (56.9)	130 (49.2)
Iwate	2,456 (37.3)	534 (33.7)	367 (33.3)	910 (37.1)	511 (43.1)	134 (50.8)

Continuous variables are shown as mean (SD) or median (IQR). Categorical variables are displayed as numbers (percentages)

Abbreviations: *BMI* body mass index; *GDM* gestational diabetes mellitus; *GEJE* Great East Japan Earthquake; *HDP* hypertensive disorders of pregnancy; *IQR* interquartile range; *SD* standard deviation; *T2DM* type 2 diabetes mellitus; *WC* waist circumference

#### Characteristics of the Postmenopausal Women by Parity

The characteristics of postmenopausal women and those according to parity are shown in Table 2. The study was conducted in Japan. Unlike premenopausal women, T2DM prevalence, mean value of current BMI, proportions of current obesity, and WC ≥ 90 cm were higher as parity increased in postmenopausal women. As parity increased, the proportions of family history of hypertension and menopause due to surgery of the uterus and/or ovary decreased. As in premenopausal women, the prevalence of hypertension increased as parity increased in postmenopausal women. As parity increased, the proportion of patients with a family history of T2DM decreased. The proportions of unmarried and divorced women were higher in nulliparous women and in women with parity of one. In addition, the lower the parity, the higher the level of education in those women.

**Table 2:** Characteristics of postmenopausal women by parity

Variables	All (*N* = 23,528)	Parity				
		0 (*N* = 1,614)	1 (*N* = 2,083)	2 (*N* = 11,447)	3 (*N* = 7,160)	≥ 4 (*N* = 1,224)
Age, years	63.9 (6.2)	61.5 (7.0)	63.3 (7.4)	64.4 (5.9)	63.9 (5.9)	63.7 (6.2)
Category of age, *N* (%)						
20–29.9 years	12 (0.1)	0 (0.0)	8 (0.4)	2 (0.0)	2 (0.0)	0 (0.0)
30–39.9 years	59 (0.3)	6 (0.4)	14 (0.7)	24 (0.2)	12 (0.2)	3 (0.2)
40–49.9 years	279 (1.2)	73 (4.5)	55 (2.6)	84 (0.7)	52 (0.7)	15 (1.2)
50–59.9 years	4,787 (20.3)	497 (30.8)	432 (20.7)	2,077 (18.1)	1,503 (21.0)	278 (22.7)
60–69.9 years	13,578 (57.7)	816 (50.6)	1,122 (53.9)	6,744 (58.9)	4,218 (58.9)	678 (55.4)
≥ 70 years	4,813 (20.5)	222 (13.8)	452 (21.7)	2,516 (22.0)	1,373 (19.2)	250 (20.4)
Waist circumference, cm	82.0 (9.1)	80.3 (9.9)	81.2 (9.5)	81.6 (8.9)	83.0 (9.0)	84.0 (9.2)
Waist circumference ≥ 90 cm, *N* (%)	4,148 (17.6)	253 (15.7)	337 (16.2)	1,830 (16.0)	1,433 (20.0)	295 (24.1)
Height, cm	152.1 (5.6)	153.3 (5.9)	152.2 (5.8)	152.0 (5.5)	152.1 (5.5)	151.5 (5.7)
Current body weight, kg	53.3 (8.6)	52.4 (9.4)	52.8 (9.1)	52.8 (8.4)	54.3 (8.5)	55.1 (8.7)
Current BMI, kg/m^2^	23.1 (3.6)	22.3 (3.7)	22.8 (3.8)	22.9 (3.5)	23.5 (3.5)	24.0 (3.6)
Category of current BMI, *N* (%)						
Underweight (< 18.5 kg/m^2^)	1,786 (7.6)	230 (14.3)	208 (10.0)	906 (7.9)	400 (5.6)	42 (3.4)
Normal range (18.5–24.9 kg/m^2^)	15,801 (67.2)	1,053 (65.2)	1,410 (67.7)	7,876 (68.8)	4,699 (65.6)	763 (62.3)
Obese (≥ 25.0 kg/m^2^)	5,941 (25.3)	331 (20.5)	465 (22.3)	2,665 (23.3)	2,061 (28.8)	419 (34.2)
Body weight at 20 years, kg	51.0 (7.4)	51.2 (8.6)	50.3 (8.2)	50.6 (6.8)	51.6 (7.6)	51.5 (7.7)
BMI at 20 years, kg/m^2^	22.0 (3.1)	21.8 (3.4)	21.7 (3.6)	21.9 (2.9)	22.3 (3.3)	22.5 (3.4)
Category of BMI at 20 years, *N* (%)						
Underweight (< 18.5 kg/m^2^)	1,396 (5.9)	129 (8.0)	184 (8.8)	695 (6.1)	319 (4.5)	69 (5.6)
Normal range (18.5–24.9 kg/m^2^)	19,595 (83.3)	1,316 (81.5)	1,697 (81.5)	9,637 (84.2)	5,970 (83.4)	975 (79.7)
Obese (≥ 25.0 kg/m^2^)	2,537 (10.8)	169 (10.5)	202 (9.7)	1,115 (9.7)	871 (12.2)	180 (14.7)
Body weight gain after 20 years, kg	2.4 (9.1)	1.2 (9.7)	2.5 (10.0)	2.2 (8.6)	2.7 (9.3)	3.6 (9.9)
Physical activity level, METS, Median (IQR)	28.2 (21.8–37.7)	27.0 (21.3–34.0)	26.8 (20.5–35.1)	28.0 (21.7–37.0)	29.0 (22.2–39.6)	31.0 (23.0–42.2)
Smoking status, *N* (%)						
Never smoker	20,550 (87.3)	1,275 (79.0)	1,724 (82.8)	10,103 (88.3)	6,389 (89.2)	1,059 (86.5)
Ever smoker	1,447 (6.2)	185 (11.5)	193 (9.3)	661 (5.8)	337 (4.7)	71 (5.8)
Current smoker	960 (4.1)	129 (8.0)	122 (5.9)	399 (3.5)	257 (3.6)	53 (4.3)
Missing	571 (2.4)	25 (1.5)	44 (2.1)	284 (2.5)	177 (2.5)	41 (3.3)
Alcohol consumption, *N* (%)						
Never drinker	15,594 (66.3)	982 (60.8)	1,418 (68.1)	7,675 (67.0)	4,685 (65.4)	834 (68.1)
Ever drinking	333 (1.4)	26 (1.6)	45 (2.2)	150 (1.3)	90 (1.3)	22 (1.8)
Current drinking	7,365 (31.3)	597 (37.0)	604 (29.0)	3,507 (30.6)	2,307 (32.2)	350 (28.6)
Missing	236 (1.0)	9 (0.6)	16 (0.8)	115 (1.0)	78 (1.1)	18 (1.5)
T2DM prevalence, *N* (%)	1,875 (8.0)	108 (6.7)	150 (7.2)	877 (7.7)	612 (8.5)	128 (10.5)
Hypertension prevalence, *N* (%)	9,726 (41.3)	560 (34.7)	801 (38.5)	4,804 (42.0)	3,032 (42.3)	529 (43.2)
Own birth weight, *N* (%)						
< 2,500 g	2,248 (9.6)	176 (10.9)	220 (10.6)	1,080 (9.4)	665 (9.3)	107 (8.7)
2,500–3,499 g	7,957 (33.8)	662 (41.0)	706 (33.9)	3,684 (32.2)	2,478 (34.6)	427 (34.9)
≥ 3,500 g	525 (2.2)	70 (4.3)	48 (2.3)	228 (2.0)	150 (2.1)	29 (2.4)
Unknown	11,577 (49.2)	647 (40.1)	993 (47.7)	5,848 (51.1)	3,503 (48.9)	586 (47.9)
Missing	1,221 (5.2)	59 (3.7)	116 (5.6)	607 (5.3)	364 (5.1)	75 (6.1)
History of thyroid dysfunction, *N* (%)						
Yes	1,337 (5.7)	98 (6.1)	104 (5.0)	668 (5.8)	402 (5.6)	65 (5.3)
No	22,045 (93.7)	1,409 (87.3)	1,974 (94.8)	10,760 (94.0)	6,746 (94.2)	1,156 (94.4)
Missing	146 (0.6)	107 (6.6)	5 (0.2)	19 (0.2)	12 (0.2)	3 (0.2)
History of endometriosis, *N* (%)						
Yes	1,090 (4.6)	155 (9.6)	157 (7.5)	507 (4.4)	243 (3.4)	28 (2.3)
No	22,323 (94.9)	1,354 (83.9)	1,925 (92.4)	10,936 (95.5)	6,913 (96.6)	1,195 (97.6)
Missing	115 (0.5)	105 (6.5)	1 (0.0)	4 (0.0)	4 (0.1)	1 (0.1)
History of mental diseases, *N* (%)						
Yes	681 (2.9)	70 (4.3)	84 (4.0)	315 (2.8)	172 (2.4)	40 (3.3)
No	22,685 (96.4)	1,434 (88.8)	1,991 (95.6)	11,108 (97.0)	6,971 (97.4)	1,181 (96.5)
Missing	162 (0.7)	110 (6.8)	8 (0.4)	24 (0.2)	17 (0.2)	3 (0.2)
Breastfeeding experience, *N* (%)						
Yes	19,151 (81.4)	0 (0.0)	1,590 (76.3)	9,832 (85.9)	6,562 (91.6)	1,167 (95.3)
No	4,181 (17.8)	1,539 (95.4)	460 (22.1)	1,564 (13.7)	566 (7.9)	52 (4.2)
Missing	196 (0.8)	75 (4.6)	33 (1.6)	51 (0.4)	32 (0.4)	5 (0.4)
Use of oral contraceptives, *N* (%)						
Yes	627 (2.7)	30 (1.9)	53 (2.5)	267 (2.3)	238 (3.3)	39 (3.2)
No	22,169 (94.2)	1,346 (83.4)	1,963 (94.2)	10,936 (95.5)	6,769 (94.5)	1,155 (94.4)
Missing	732 (3.1)	238 (14.7)	67 (3.2)	244 (2.1)	153 (2.1)	30 (2.5)
Use of hormone replacement therapy, *N* (%)						
Yes	1,802 (7.7)	126 (7.8)	177 (8.5)	903 (7.9)	497 (6.9)	99 (8.1)
No	21,131 (89.8)	1,306 (80.9)	1,855 (89.1)	10,342 (90.3)	6,531 (91.2)	1,097 (89.6)
Missing	595 (2.5)	182 (11.3)	51 (2.4)	202 (1.8)	132 (1.8)	28 (2.3)
≥ 15 years at menarche, *N* (%)						
< 15 years	18,171 (77.2)	1,360 (84.3)	1,603 (77.0)	8,726 (76.2)	5,540 (77.4)	942 (77.0)
≥ 15 years	5,121 (21.8)	242 (15.0)	455 (21.8)	2,608 (22.8)	1,551 (21.7)	265 (21.7)
Missing	236 (1.0)	12 (0.7)	25 (1.2)	113 (1.0)	69 (1.0)	17 (1.4)
≥ 35 years at last delivery, *N* (%)						
< 35 years	19,412 (82.5)	0 (0.0)	1,721 (82.6)	10,631 (92.9)	6,209 (86.7)	851 (69.5)
≥ 35 years	2,084 (8.9)	0 (0.0)	275 (13.2)	622 (5.4)	837 (11.7)	350 (28.6)
Missing	2,032 (8.6)	1,614 (100.0)	87 (4.2)	194 (1.7)	114 (1.6)	23 (1.9)
Menstrual cycle, *N* (%)						
Regular	18,150 (77.1)	1,210 (75.0)	1,510 (72.5)	8,837 (77.2)	5,654 (79.0)	939 (76.7)
Irregular	3,917 (16.6)	337 (20.9)	415 (19.9)	1,936 (16.9)	1,048 (14.6)	181 (14.8)
Missing	1,461 (6.2)	67 (4.2)	158 (7.6)	674 (5.9)	458 (6.4)	104 (8.5)
History of GDM, *N* (%)	33 (0.1)	0 (0.0)	6 (0.3)	17 (0.1)	8 (0.1)	2 (0.2)
History of HDP, *N* (%)						
Yes	1,013 (4.3)	0 (0.0)	109 (5.2)	537 (4.7)	316 (4.4)	51 (4.2)
No	22,503 (95.6)	1,614 (100.0)	1,972 (94.7)	10,905 (95.3)	6,840 (95.5)	1,172 (95.8)
Missing	12 (0.1)	0 (0.0)	2 (0.1)	5 (0.0)	4 (0.1)	1 (0.1)
Family history of T2DM, *N* (%)						
Yes	2,476 (10.5)	250 (15.5)	227 (10.9)	1,193 (10.4)	697 (9.7)	109 (8.9)
No	20,928 (88.9)	1,275 (79.0)	1,850 (88.8)	10,239 (89.4)	6,451 (90.1)	1,113 (90.9)
Missing	124 (0.5)	89 (5.5)	6 (0.3)	15 (0.1)	12 (0.2)	2 (0.2)
Family history of hypertension, *N* (%)						
Yes	8,541 (36.3)	761 (47.1)	784 (37.6)	4,176 (36.5)	2,447 (34.2)	373 (30.5)
No	14,929 (63.5)	812 (50.3)	1,297 (62.3)	7,266 (63.5)	4,705 (65.7)	849 (69.4)
Missing	58 (0.2)	41 (2.5)	2 (0.1)	5 (0.0)	8 (0.1)	2 (0.2)
Marital status, *N* (%)						
Married	18,489 (78.6)	707 (43.8)	1,552 (74.5)	9,329 (81.5)	5,918 (82.7)	983 (80.3)
Unmarried	845 (3.6)	687 (42.6)	27 (1.3)	69 (0.6)	50 (0.7)	12 (1.0)
Divorced	977 (4.2)	68 (4.2)	203 (9.7)	443 (3.9)	216 (3.0)	47 (3.8)
Widowed	3,063 (13.0)	140 (8.7)	290 (13.9)	1,530 (13.4)	937 (13.1)	166 (13.6)
Missing	154 (0.7)	12 (0.7)	11 (0.5)	76 (0.7)	39 (0.5)	16 (1.3)
Highest level of education, *N* (%)						
Low	5,272 (22.4)	186 (11.5)	456 (21.9)	2,471 (21.6)	1,733 (24.2)	426 (34.8)
Medium	15,358 (65.3)	1,066 (66.0)	1,347 (64.7)	7,691 (67.2)	4,600 (64.2)	654 (53.4)
High	2,638 (11.2)	348 (21.6)	257 (12.3)	1,165 (10.2)	741 (10.3)	127 (10.4)
Missing	260 (1.1)	14 (0.9)	23 (1.1)	120 (1.0)	86 (1.2)	17 (1.4)
Breakfast skipping, *N* (%)						
Not breakfast skipping	21,701 (92.2)	1,432 (88.7)	1,890 (90.7)	10,612 (92.7)	6,644 (92.8)	1,123 (91.7)
Breakfast skipping	1,388 (5.9)	166 (10.3)	158 (7.6)	615 (5.4)	377 (5.3)	72 (5.9)
Missing	439 (1.9)	16 (1.0)	35 (1.7)	220 (1.9)	139 (1.9)	29 (2.4)
Average sleeping time/day, *N* (%)						
< 7 h	17,401 (74.0)	1,171 (72.6)	1,569 (75.3)	8,516 (74.4)	5,281 (73.8)	864 (70.6)
≥ 7 and < 8 h	4,716 (20.0)	335 (20.8)	400 (19.2)	2,266 (19.8)	1,457 (20.3)	258 (21.1)
≥ 8 h	1,388 (5.9)	106 (6.6)	113 (5.4)	654 (5.7)	415 (5.8)	100 (8.2)
Missing	23 (0.1)	2 (0.1)	1 (0.0)	11 (0.1)	7 (0.1)	2 (0.2)
Nap time, *N* (%)						
Not taking a nap	14,281 (60.7)	1,092 (67.7)	1,302 (62.5)	7,099 (62.0)	4,105 (57.3)	683 (55.8)
Nap time is < one hour/day	7,626 (32.4)	390 (24.2)	620 (29.8)	3,584 (31.3)	2,588 (36.1)	444 (36.3)
Nap time is ≥ one hour/day	1,512 (6.4)	125 (7.7)	152 (7.3)	719 (6.3)	432 (6.0)	84 (6.9)
Missing	109 (0.5)	7 (0.4)	9 (0.4)	45 (0.4)	35 (0.5)	13 (1.1)
Number of relocations after the GEJE, *N* (%)						
0	18,146 (77.1)	1,208 (74.8)	1,571 (75.4)	8,877 (77.5)	5,553 (77.6)	937 (76.6)
1	1,367 (5.8)	133 (8.2)	118 (5.7)	665 (5.8)	381 (5.3)	70 (5.7)
2	991 (4.2)	89 (5.5)	101 (4.8)	461 (4.0)	292 (4.1)	48 (3.9)
3	961 (4.1)	70 (4.3)	104 (5.0)	441 (3.9)	311 (4.3)	35 (2.9)
≥ 4	510 (2.2)	38 (2.4)	58 (2.8)	256 (2.2)	133 (1.9)	25 (2.0)
Missing	1,553 (6.6)	76 (4.7)	131 (6.3)	747 (6.5)	490 (6.8)	109 (8.9)
Year, *N* (%)						
2013	3,988 (17.0)	321 (19.9)	370 (17.8)	1,815 (15.9)	1,197 (16.7)	285 (23.3)
2014	10,826 (46.0)	778 (48.2)	972 (46.7)	5,349 (46.7)	3,232 (45.1)	495 (40.4)
2015	8,714 (37.0)	515 (31.9)	741 (35.6)	4,283 (37.4)	2,731 (38.1)	444 (36.3)
Prefecture, *N* (%)						
Miyagi	12,463 (53.0)	852 (52.8)	1,058 (50.8)	6,256 (54.7)	3,789 (52.9)	508 (41.5)
Iwate	11,065 (47.0)	762 (47.2)	1,025 (49.2)	5,191 (45.3)	3,371 (47.1)	716 (58.5)
Menopause age, *N* (%)						
Premature menopause (Age at menopause was < 40 years)	902 (3.8)	103 (6.4)	139 (6.7)	418 (3.7)	206 (2.9)	36 (2.9)
Postmenopause (Age at menopause was ≥ 40 years)	22,082 (93.9)	1,478 (91.6)	1,887 (90.6)	10,789 (94.3)	6,774 (94.6)	1,154 (94.3)
Missing	544 (2.3)	33 (2.0)	57 (2.7)	240 (2.1)	180 (2.5)	34 (2.8)
Reasons of menopause, *N* (%)						
Natural menopause	19,051 (81.0)	1,213 (75.2)	1,543 (74.1)	9,317 (81.4)	5,966 (83.3)	1,012 (82.7)
Menopause due to surgery of uterus and/or ovary	3,424 (14.6)	298 (18.5)	394 (18.9)	1,692 (14.8)	897 (12.5)	143 (11.7)
Other reasons	757 (3.2)	64 (4.0)	116 (5.6)	326 (2.8)	205 (2.9)	46 (3.8)
Missing	296 (1.3)	39 (2.4)	30 (1.4)	112 (1.0)	92 (1.3)	23 (1.9)

Continuous variables are shown as mean (SD) or median (IQR). Categorical variables are presented as numbers (percentages)

Abbreviations: *BMI* body mass index; *GDM* gestational diabetes mellitus; *GEJE* Great East Japan Earthquake; *HDP* hypertensive disorders of pregnancy; *IQR* interquartile range; *SD* standard deviation; *T2DM* type 2 diabetes mellitus; *WC* waist circumference

#### The Association of Parity with T2DM in Premenopausal Women (Models 1–5)

**Fig. 2:**
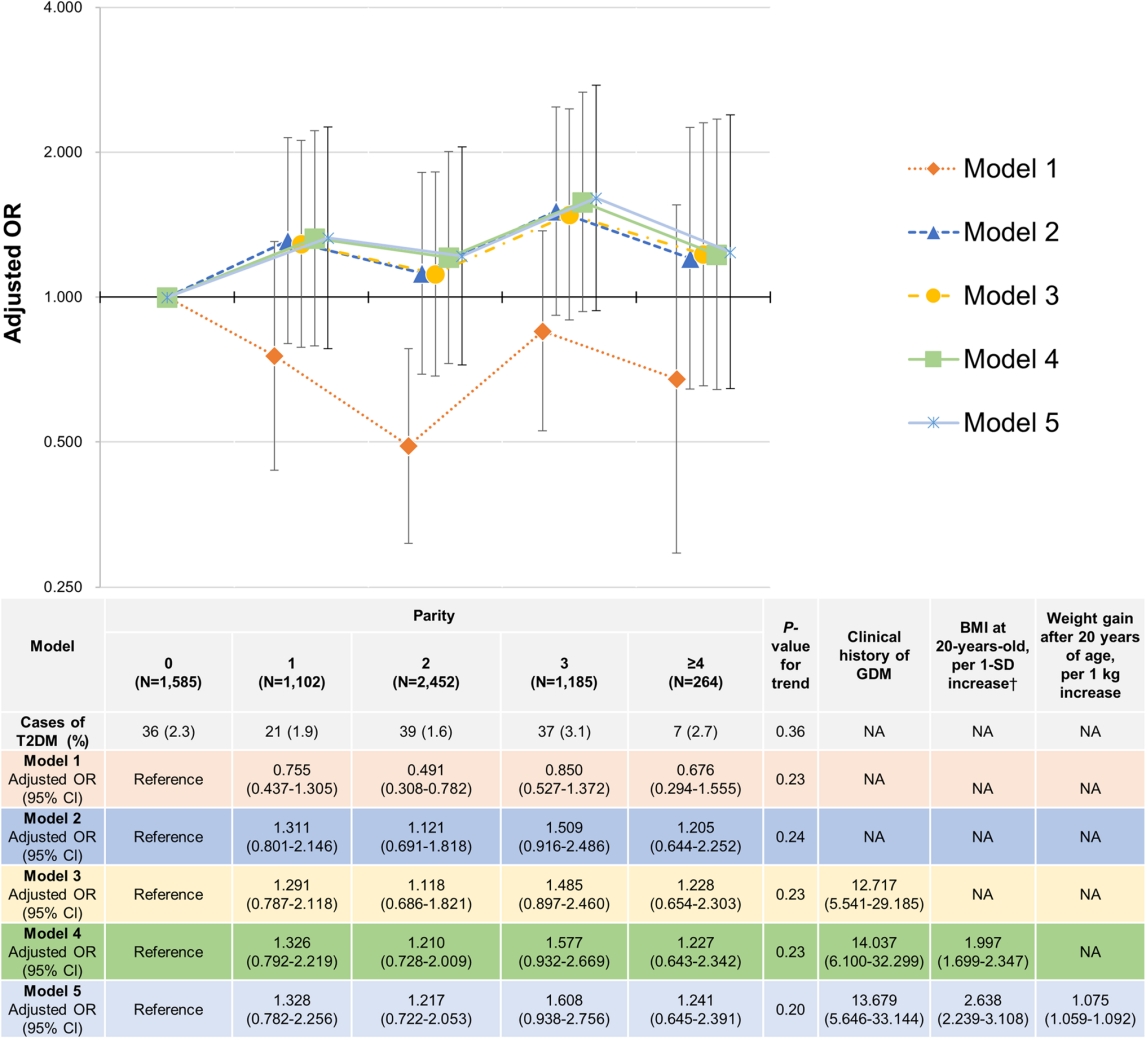
Association of parity with T2DM in premenopausal women (Models 1–5). †1-SD value was 3.0 kg/m^2^ for BMI at 20-years-old. Model 1: Adjusting for age. Model 2: Model 1 plus covariates as follows: height, physical activity, marital status, smoking status, alcohol consumption, own birth weight, highest educational level, family history of T2DM, family history of hypertension, breastfeeding experience, use of oral contraceptives, use of hormone replacement therapy, thyroid dysfunction, endometriosis, mental disease, menstrual cycle, age at menarche (< 15 years or ≥ 15 years), age at last delivery (< 35 years or ≥ 35 years), sleeping time, nap time, year of study participation, Prefecture (Miyagi or Iwate), and number of relocations after the GEJE. Model 3: Model 2 plus a clinical history of GDM. Model 4: Model 3 plus BMI at 20-years-old, as per 1-SD increase. Model 5: Model 4 plus weight gain after 20 years of age, as per 1 kg increase. Abbreviations: *BMI* body mass index; *CI* confidence interval; *DM* diabetes mellitus; *GDM* gestational diabetes mellitus; *GEJE* great East Japan earthquake; *OR* odds ratio; *SD* standard deviation; *T2DM* type 2 diabetes mellitus; *NA* not applicable

The results of the association between parity and T2DM in premenopausal women in Models 1–5 are shown in Fig. 2. In Model 1, women with a parity of two had lower odds of developing T2DM than nulliparous women. In women of other parities, the association between parity and T2DM was not statistically significant. In addition, no significant linear association was observed between parity and T2DM (*P*-value for trend was 0.23). Similarly, in Models 2 and 3, no significant linear association between parity and T2DM was observed (*P*-values for trend were 0.24 and 0.23 in Models 2 and 3, respectively). In Model 3, women with a clinical history of GDM had higher odds for T2DM than those without a history of GDM. The adjusted odds ratio (OR) was 12.717 (95% confidence interval [CI]:5.541–29.185). No significant linear association between parity and T2DM was observed (*P*-value for trend was 0.23) in Model 4. Women with a clinical history of GDM had significantly higher odds of developing T2DM, with an adjusted OR of 14.037 (95% CI:6.100–32.299). BMI at 20-years-old was also significantly associated with T2DM, and the adjusted OR for each 1-SD increase in BMI at 20-years-old was 1.997 (95% CI:1.699–2.347). Further, no significant linear association between parity and T2DM was observed (*P*-value for trend was 0.20) in Model 5. Compared with women without a clinical history of GDM, in those with a clinical history of GDM, the OR for T2DM was significantly higher with an adjusted OR of 13.679 (95% CI:5.646–33.144). BMI at 20-years-old was also significantly related to T2DM, and the adjusted OR for each 1-SD increase in BMI at 20 years of age was 2.638 (95% CI:2.239–3.108). Weight gain after 20 years of age was related to T2DM. The adjusted OR per 1 kg increase in weight gain after 20 years of age was 1.075 (95% CI:1.059–1.092).

#### The Association of Parity with T2DM In Postmenopausal Women (Models 1–5)

**Fig. 3:**
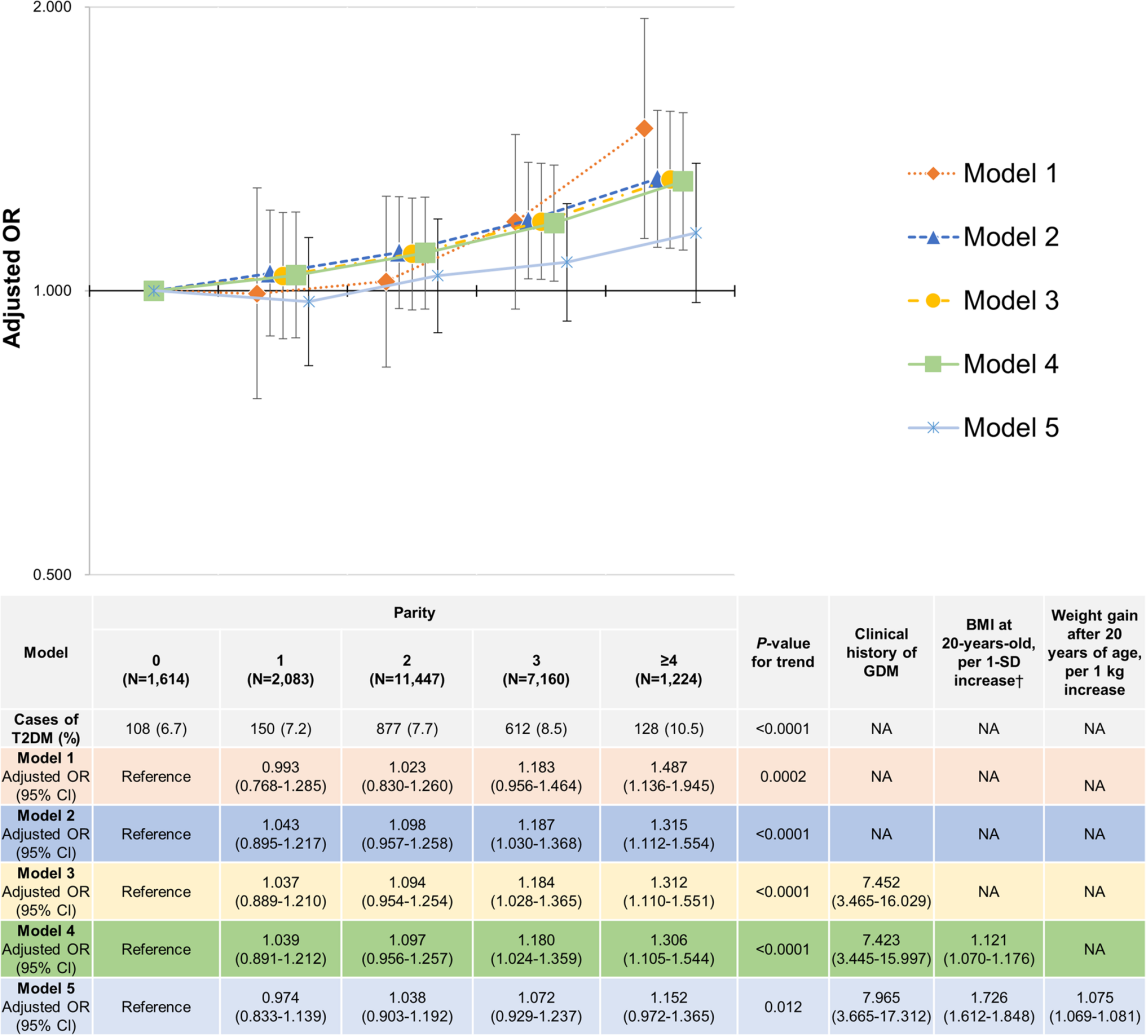
Association of parity with T2DM in postmenopausal women (Models 1–5). †1-SD value was 3.1 kg/m^2^ for BMI at 20 years old. Model 1: Adjusting for age. Model 2: Model 1 plus covariates as follows: height, physical activity, marital status, smoking status, alcohol consumption, own birth weight, highest educational level, family history of hypertension, breastfeeding experience, use of oral contraceptives, use of hormone replacement therapy, thyroid dysfunction, endometriosis, mental disease, menstrual cycle, age at menarche (< 15 years or ≥ 15 years), age at last delivery (< 35 years or ≥ 35 years), menopause age (< 40 years or ≥ 40 years), sleeping time, nap time, year of study participation, Prefecture (Miyagi or Iwate), and number of relocations after the GEJE. Model 3: Model 2 plus a clinical history of GDM. Model 4: Model 3 plus BMI at 20-years-old, as per 1-SD increase. Model 5: Model 4 plus weight gain after 20 years of age as per 1 kg increase. Abbreviations: *BMI* body mass index; *CI* confidence interval; *DM* diabetes mellitus; *GDM* gestational diabetes mellitus; *GEJE* great East Japan earthquake; *OR* odds ratio, *SD* standard deviation; *T2DM* type 2 diabetes mellitus; *NA* not applicable

Figure 3 depicts the results of the association between parity and T2DM in Models 1–5 in postmenopausal women. In Models 1 and 2, the odds of T2DM increased as the number of parities increased. A significant linear association between parity and T2DM was observed (*P* values for trend were 0.0002 and < 0.0001 in Models 1 and 2, respectively). Model 3 also exhibited a significant linear association between higher parity and higher odds of T2DM (*P*-value for trend < 0.0001). Women with a clinical history of GDM had significantly higher odds of T2DM compared with those without a clinical history of GDM, with an adjusted OR of 7.452 (95% CI:3.465–16.029). In Model 4, a significant linear association between parity and T2DM remained (*P*-value for trend < 0.0001). A clinical history of GDM was associated with a high risk of T2DM. The adjusted OR for T2DM was 7.423 (95% CI:3.445–15.997). BMI at 20 years was also related to T2DM. The adjusted OR for each 1-SD increase in BMI at 20-years-old was 1.121 (95% CI:1.070–1.176). In Model 5, the association between parity and T2DM was attenuated compared to the results of Model 4, although a significant linear association remained (*P*-value for trend was 0.012). For women with a clinical history of GDM, the odds of T2DM were significantly higher than that for those without a clinical history of GDM, with an adjusted OR of 7.965 (95% CI:3.665–17.312). BMI at 20-years-old was also significantly related to T2DM, and the adjusted OR for each 1-SD increase in BMI at 20-years-old was 1.726 (95% CI:1.612–1.848). Weight gain after 20 years of age was also related to T2DM, with an adjusted OR of 1.075 (95% CI:1.069–1.081) per 1 kg increase.

#### The Association of Parity with T2DM (Models 6 and 7)

As additional analysis, Model 6 was created by adjusting for current BMI, as an indicator of current overall adiposity, in addition to Model 3. Model 7 was further created by adjusting for current WC, as an indicator of current abdominal adiposity, in addition to Model 3. The results of Models 6 and 7 in premenopausal women are shown in Supplementary Fig. S1 and Supporting Information. The results in postmenopausal women are shown in Supplementary Fig. S2 and Supporting Information.

#### Combined Analysis for Investigation of Interaction Between a Clinical History of GDM and Parity in Multiparous Women

The results of the combined analysis for interaction between a clinical history of GDM and parity in premenopausal multiparous women are shown in Supplementary Fig. S3 and Supporting Information. The results in postmenopausal women are shown in Supplementary Fig. S4 and Supporting Information.

#### Associations of Parity with T2DM with Women of Parity = 1 Set as a Reference Category

The results of the association between parity and T2DM in premenopausal women when women with parity of one was set as a reference category are shown in Supplementary Fig. S5 and Supporting Information. The results in postmenopausal women are shown in Supplementary Fig. S6 and Supporting Information.

#### Associations of Parity with T2DM Stratified by Age

The results of the association between parity and T2DM in women aged 50–64 years are shown in Supplementary Fig. S7 and Supporting Information. The results of the association between parity and T2DM in women aged ≥ 65 years are shown in Supplementary Fig. S8 and Supporting Information.

## Discussion

This is the first study to examine the association between parity and T2DM, considering the clinical history of GDM according to the current menstrual status in Japan. In premenopausal women, increased parity was not significantly associated with T2DM. In contrast, in postmenopausal women, an increase in parity was associated with the risk of T2DM, and this association was attenuated when adjusting for several adiposity indices.

Several reasons for the difference in the association between parity and T2DM, depending on menopausal status were considered. It is assumed that some of the premenopausal women would have more childbirths in the future, which would not necessarily reflect the lifetime parity count of a given woman. Furthermore, since the mean age of premenopausal women was lower than that of postmenopausal women, a lower T2DM prevalence was observed. Further, as age is one of the risk factors for T2DM, some premenopausal women may develop T2DM after menopause, after which the association between parity and T2DM may become apparent [29].

A meta-analysis shows a linear graded association between parity and the risk of T2DM, although Japanese women were not included in the analysis [10]. In line with their results, our study demonstrates a consistently statistically significant association between parity and T2DM, although the strength of the association was attenuated after adjusting for BMI at 20 years and weight-gain after adulthood, current BMI, or current WC in our study (the results of adjustment with current BMI or WC are shown in Supplementary Fig. S2). However, our results are inconsistent with those of previous studies. Another cohort study in Japan reported that the association between parity and increasing T2DM risk diminished after adjusting for BMI [17]. Differences in the definition of T2DM, the number of T2DM patients, covariates, and presence or absence of stratification in current menopausal status between their study and our study might have led to different results. In addition, Manson et al*.* reported that the association between parity and T2DM risk diminished after adjusting for weight gain after adulthood in the US [30]. Differences in ethnicity, lifestyle, and covariates may have resulted in different results between their study and ours.

In postmenopausal women, parity was associated with increased insulin resistance, even after adjusting for BMI [31]. High parity, accompanied by high estrogen exposure, may result in long cumulative exposure to insulin resistance, because insulin sensitivity decreases during pregnancy and high estrogen levels [32]. Since pancreatic β-cells are key regulators of glucose homeostasis, impairment of β-cells due to an increase in insulin resistance is known to be central to the development of DM [10].

The attenuation of the association between parity and T2DM after adjusting for weight-gain after 20 years of age, or current BMI, or WC in postmenopausal women in our study may be partly explained by previous studies. Long-term weight-gain is a risk factor of T2DM [27]. Additionally, prolonged obesity may impair insulin secretion and increase resistance to glucose uptake [33]. Obesity-related insulin resistance results in more rapid fat accumulation during pregnancy and changes fat distribution characterized by higher weight gain and greater abdominal fat deposition [32]. Even after adjusting for a clinical history of GDM, a positive and graded relationship between parity and risk of developing DM was reported in a prospective study in Canada [34], which is consistent with the results on postmenopausal women in our study. Both premenopausal and postmenopausal women with a clinical history of GDM were at a high risk of T2DM in our study. Furthermore, our study showed that the association between a clinical history of GDM and T2DM did not attenuate even after adjusting for the adiposity index. The results of the association between a clinical history of GDM and T2DM in our study were in line with those of other studies. Vounzoulaki et al. reported in a meta-analysis that women with a clinical history of GDM are at nearly tenfold higher risk of developing T2DM than those without a clinical history of GDM [35]. GDM is suggested to be the result of pancreatic β-cell dysfunction due to chronic insulin resistance during pregnancy. As the dysfunction could be progressive after delivery, GDM is associated with a high risk of T2DM [36].

The strength of our study is that we considered several covariates, including medical history, lifestyle habits, psychiatric history, and social factors in the statistical analysis, since our study included a large sample size of women. However, our study has several limitations. Information on the parity count was obtained from questionnaires, which may have led to misclassifications. However, based on previous studies, the number of children written in self-reported questionnaires almost matches the number of children in the medical records; thus, this limitation was not considered to have a critical impact on the results of this study [37]. We did not perform blood test twice, and the multiversity of situations used in the definition of T2DM may have led to misclassification of T2DM. Hormonal measurements were not taken to confirm menopause, which may have led to misclassification of premenopausal and postmenopausal women. Because our study is cross-sectional, a causal relationship between parity and T2DM could not be proven. Therefore, prospective cohort studies are warranted. Furthermore, despite the fact that the risk of developing GDM is higher in women with multiple pregnancies than women with single parity because of increased placental hormones and additional stress on the body and higher demand for insulin [38], information on multiple pregnancies and stillbirth were not collected in this study, limited the consideration of its association with GDM.

In conclusion, parity is associated with an increased risk of developing T2DM in postmenopausal women but not in premenopausal women. In postmenopausal women, maintaining an appropriate body weight would be beneficial in attenuating the increased risk for T2DM. A clinical history of GDM is a risk factor for T2DM in both pre- and postmenopausal women; therefore, women with a clinical history of GDM require continuous medical checkups to screen for T2DM. Our findings underscore the importance of prioritizing health among women with multiple childbirths, who may experience heightened stress and reduced self-care during child-rearing [39]. Enhanced awareness and proactive health management are essential for this demographic.

## Supplementary Information

Below is the link to the electronic supplementary material.

Supporting Information and Supplementary Table S1. Differences in characteristics between women who were analyzed and excluded owing to missing data or clinically improbable data (PDF 337 KB)

Supplementary Fig S1. Association of parity with T2DM in premenopausal women (Models 6 and 7). Supplementary Fig S2. Association of parity with T2DM in postmenopausal women (Models 6 and 7) (PDF 128 KB)

Supplementary Fig S3. Combined analysis for investigation of interaction between a clinical history of GDM and parity in premenopausal women. Supplementary Fig S4. Combined analysis for investigation of interaction between a clinical history of GDM and parity in postmenopausal women (PDF 127 KB)

Supplementary Fig S5. Association of parity with T2DM in premenopausal women (Women with parity=1 set as a reference category). Supplementary Fig S6. Association of parity with T2DM in postmenopausal women (Women with parity=1 set as a reference category) (PDF 134 KB)

Supplementary Fig S7. Association of parity with T2DM in women aged 50 to 64. Supplementary Fig S8. Association of parity with T2DM in women aged 65 or more (PDF 141 KB)

## Data Availability

The data that support the findings of this study are available upon reasonable request and following procedures with permission of dist@megabank.tohoku.ac.jp.
