# Supplementary material for: Association of Parity with Type 2 Diabetes Mellitus in Japan
Source: Reprod Sci. 2024 Dec 11;32(2):366–81. doi: 10.1007/s43032-024-01752-z (PMC11825537; doi:10.1007/s43032-024-01752-z)
Supplement: Supplementary file 1 — Supporting Information and Supplementary Table S1. Differences in characteristics between women who were analyzed and excluded owing to missing data or clinically improbable data (PDF 337 KB) [file 43032_2024_1752_MOESM1_ESM.pdf]

SUPPORTING INFORMATION

Association of Parity with Type 2 Diabetes Mellitus in Japan

Hongxin Wang[a], Noriyuki Iwama[a,b,c], Keiichi Yuwaki[d], You Nakamichi[d], Hirotaka Hamada[a], Hasumi Tomita[a], Kazuma Tagami[a], Rie Kudo[a], Natsumi Kumagai[a], Hirohito Metoki[c,e], Naoki Nakaya[c], Atsushi Hozawa[c], Shinichi Kuriyama[f,g,h], Nobuo Yaegashi[a,b,c,h], and Masatoshi Saito[a,b,i]

[a] Department of Obstetrics and Gynecology, Tohoku University Graduate School of Medicine, 1-1, Seiryomachi, Sendai 980-8574, Miyagi, Japan

[b] Women's Health Care Medical Science, Tohoku University Graduate School of Medicine, 1-1, Seiryomachi, Sendai 980-8574, Miyagi, Japan

[c] Tohoku Medical Megabank Organization, Tohoku University, 2-1, Seiryomachi, Sendai 980-8573, Miyagi, Japan

[d] Underwriting and Medical Department, The Dai-ichi Life Insurance Company, Limited, Koto-ku, Tokyo, Japan

[e] Division of Public Health, Hygiene and Epidemiology, Tohoku Medical Pharmaceutical University, 1-15-1 Fukumuro, Sendai 983-8536, Miyagi, Japan

[f] Division of Molecular Epidemiology, Tohoku University Graduate School of Medicine, 1-1, Seiryomachi, Sendai 980-8574, Miyagi, Japan

[g] International Research Institute of Disaster Science, Tohoku University, 468-1, Aramaki, Sendai 980-8572, Miyagi, Japan

[h] Environment and Genome Research Center, Tohoku University Graduate School of Medicine, 2-1, Seiryomachi, Sendai 980-8575, Miyagi, Japan Sendai, Miyagi, Japan

[I] Department of Maternal and Fetal Therapeutics, Tohoku University Graduate School of Medicine, 1-1, Seiryomachi, Sendai 980-8574, Miyagi, Japan

Corresponding Author

Noriyuki Iwama, MD, PhD

Department of Obstetrics and Gynecology, Tohoku University Hospital

1-1, Seiryomachi, Sendai 980-8574, Miyagi, Japan

Phone: +81-22-717-7251; Fax: +81-22-717-7258

Email: noriyuki.iwama.a3@ tohoku.ac.jp

1. Collection method of other variables used in this study

Information on age, height, current body weight (BW), and current waist circumference (WC) were collected from the municipal health check-ups. BW at 20-years-old was also collected from the

self-reported questionnaire. BMI at 20-years-old was calculated as the BW in kilograms at 20-years-old divided by the square of the height in meters. BMI at 20-year-old was classified into underweight (BMI <18.5 kg/m^2), normal range (≥18.5 and <25.0 kg/m^2), and obese (≥25.0 kg/m^2), based on the Western Pacific Region of World Health Organization criteria for Japanese individuals (1, 2). Based on current BW and height, current underweight, normal range, and obese were defined in the same manner as BMI at 20-years-old. Weight gain after 20 years of age was calculated as follows: current BW − BW at 20 years of age. Information on marital status, smoking status, alcohol consumption, own birth weight, highest educational level, family history of type 2 diabetes mellitus (T2DM) or hypertension, breastfeeding experience, use of oral contraceptives, use of hormone replacement therapy, hyperthyroidism, hypothyroidism, endometriosis, depression before the Great East Japan Earthquake (GEJE), depression after the GEJE, bipolar disorder, schizophrenia, menstrual cycle, age at menarche, age at last delivery, average sleeping time/day, nap time, year of study participation, Prefecture (Miyagi or Iwate), and number of relocations after the GEJE were collected from the self-reported questionnaire.

Marital status was classified into four categories: married, unmarried, divorced, and widowed. We divided smoking status into three categories: never smokers, ever smokers, and current smokers. Participants who answered not smoking more than 100 cigarettes in their lifetime were defined as never smokers. Participants who answered smoking more than 100 cigarettes in their lifetime and those who were currently smoking were defined as current smokers. Participants who answered having smoked more than 100 cigarettes during their lifetime and who were currently not smoking were defined as ever smokers. Alcohol consumption status was classified into three categories: never drinkers, ever drinkers, and current drinkers. Participants who reported currently consuming alcohol were defined as current drinkers. Participants who reported having quit alcohol were defined as ever drinkers. Participants who reported rarely drinking or not able to drink were defined as never drinkers. Participants' own birth weight was categorized as follows: <2,500 g, ≥2,500 g and <3,500 g, ≥3,500 g, and unknown. We divided the variable on highest educational level attainment into three categories: low, medium, and high. With reference to previous studies (3), participants who answered that they graduated from elementary school or junior high school were defined as low, those who answered graduated from high school or vocational school were defined as medium, and those who answered graduated from college, technical college, university, or graduate school were defined as high. Participants who answered others were categorized as missing data. Family history of T2DM was defined as a history of T2DM of father, mother, brother, or sister. Thyroid dysfunction was defined as hyperthyroidism or hypothyroidism. Mental disease was defined as depression before the GEJE, depression after the GEJE, bipolar disorder, or schizophrenia. With reference to the previous study, metabolic equivalents (METs) assigned to physical activity were calculated from the information on the questionnaire to quantify the amount of physical activity (3). Delayed menarche was defined as the age at menarche ≥15 years according to the Japan Society of Obstetrics and Gynecology (4). Advanced maternal age at the last delivery was defined as age at the last delivery of ≥35 years. Skipping breakfast was defined when the frequency of breakfast was <once/month,

once-three times/month, once-twice/week, three-four times/week, or five-six times/week. Not skipping breakfast was also defined as those who ate breakfast daily. The average sleeping time/day was divided into three categories: <7 hours, ≥7 and <8 hours, and ≥8 hours (5). We divided nap time into three categories: not taking a nap, nap time is <one hour/day, and nap time is ≥one hour/day. In postmenopausal women, premature menopause was defined as menopause age of <40 years. Reasons for menopause were divided into three categories: natural menopause, menopause due to surgery of uterus and/or ovary, and menopause due to other reasons. Year of study participation was classified over three years: 2013, 2014, and 2015. Number of relocations after the GEJE was divided into five categories as follows: 0, 1, 2, 3, and ≥4.

2. Differences in characteristics between women who were analyzed and excluded owing to missing data or clinically improbable data

As shown in Supplementary Table S1, the differences in characteristics between women who were analyzed and those who were excluded, owing to missing data or clinically improbable data, were tested using Student's t-test or chi-squared test. No significant differences were observed in the prevalence of T2DM and hypertension between women who were analyzed and those that were excluded. Women who were excluded had higher proportions of obesity, low level of education, more participants from year 2013, and more Miyagi Prefecture residents than those who were analyzed. Additionally, women who were excluded had lower proportions of nulliparous women, were with higher proportions of the underweight category, and had higher proportions of those with average sleeping time/day <7 hours, compared to those who were analyzed.

3. Additional analysis

3.1 The association of parity with T2DM (Models 6 and 7)

As additional analysis, Model 6 was created by adjusting for current BMI, as an indicator of current overall adiposity, in addition to Model 3 (6). Current BMI, as per 1-SD increase, was included in Model 6. Model 7 was further created by adjusting for current WC, as an indicator of current abdominal adiposity, in addition to Model 3. Current WC, as per 1-standard deviation (SD) increase, was included in Model 7.

The results of Models 6 and 7 in premenopausal women are shown in Supplementary Fig S1. In premenopausal women, Model 6 showed no statistically significant association between parity and T2DM. Further, no statistically significant linear association between parity and T2DM was observed (*P*-value for trend was 0.20). Women with a history of gestational diabetes mellitus (GDM) had significantly higher odds for T2DM, with an adjusted odds ratio [OR] of 12.915 (95% confidence interval [CI]: 5.324–31.334), compared to those without a history of GDM. Current BMI was also significantly associated with T2DM. The adjusted OR for each 1-standard deviation (SD) increase in current BMI was 2.105 (95% CI: 1.866–2.376). Model 7 yielded no statistically significant association between parity and

T2DM. Additionally, no statistically significant linear association between parity and T2DM was observed (*P*-value for trend was 0.20). For women with a history of GDM, the odds for T2DM were significantly higher than those without a history of GDM. The adjusted OR was 12.534 (95% CI: 5.243–29.966). Current WC was significantly related to T2DM. The adjusted OR for each 1-SD increase in current WC was 2.417 (95% CI: 2.098–2.785).

The results of Models 6 and 7 in postmenopausal women are shown in Supplementary Fig S2. In Model 6, the association between parity and T2DM was attenuated compared to the results of Model 3, although a statistically significant linear association remained (*P*-value for trend was 0.012). For participants with a history of GDM, the odds of T2DM were significantly higher than those without a history of GDM, with an adjusted OR of 8.008 (95% CI: 3.679–17.432). Current BMI was also related to T2DM, and the adjusted OR for every 1-SD increase in current BMI was 1.773 (95% CI: 1.695–1.854). In Model 7, the association between parity and T2DM was attenuated compared to the results of Model 3, although a significant linear association remained (*P*-value for trend was: 0.013). For participants with a history of GDM, the odds for T2DM were significantly higher than those without a history of GDM, with an adjusted OR of 8.055 (95% CI: 3.730–17.395). Current WC was significantly related to T2DM, and the adjusted OR for each 1-SD increase in current WC was 1.812 (95% CI: 1.728–1.901).

3.2. Combined analysis for investigation of interaction between a clinical history of GDM and parity in multiparous women

Combined analysis for investigation of the interaction between a clinical history of GDM and parity in multiparous women (i.e., parity ≥1) was conducted. Parity was divided into two categories: one and ≥two. In this analysis, women with both parity of one and no clinical history of GDM were set as a reference category. Four models (namely, A, B, C, and D) were defined. In premenopausal women, Model A was adjusted for age. Model B was adjusted for the same covariates used in Model 2. Model C was adjusted for BMI at 20 years of age, as per 1-SD increase, in addition to Model B. Model D was adjusted for weight gain after 20 years of age, as per 1 kg increase, in addition to Model C. In postmenopausal women, Model A was adjusted for age. Model B was adjusted for the same covariates used in Model 2 In addition to model A. Model C was adjusted for BMI at 20 years of age, as per 1-SD increase, in addition to Model B. Model D was adjusted for weight gain after 20 years of age, as per 1-kg increase, in addition to Model C.

The results of the combined analysis for interaction between a clinical history of GDM and parity (one or ≥two) in premenopausal multiparous women are shown in Supplementary Fig S3. In all of Models A, B, C, and D, the interaction between a clinical history of GDM and parity (1 or ≥2) was not statistically significant. *P*-values for interaction were 0.63, 0.96, 0.97, and 0.84 in Models A, B, C, and D, respectively.

The results of the combined analysis for interaction between a clinical history of GDM and parity in postmenopausal women are shown in Supplementary Fig S4. The interaction between a clinical

history of GDM and parity was not statistically significant in Models A, B, C, and D. *P*-values for interaction were 0.15, 0.26, 0.16, and 0.25 in Models A, B, C, and D, respectively. The interaction between a clinical history of GDM and parity was not statistically significant, even if parity was to be classified as <2 or ≥3, or <3 or ≥4 in Models A, B, C, and D (Data not shown).

3.3. Associations of parity with T2DM with women of parity=1 set as a reference category

Considering the possibility that nulliparous women may had a medical and/or socio-economic background that forced them not to give birth or may have chosen not to deliver due to their own policy, there may be differences in baseline characteristics between nulliparous and multiparous women. Therefore, we also performed additional analyses by setting women with parity=1 as a reference category in both premenopausal and postmenopausal women in Models 1–5.

The results of the association between parity and T2DM in premenopausal women when women with parity of one was set as a reference category are shown in Supplementary Fig S5. In all Models, the association of parity with T2DM was not statistically significant. Women with a clinical history of GDM had significantly higher odds of T2DM in Models 3–5.

The results of the association between parity and T2DM in postmenopausal women when women with parity of one was set as a reference category are shown in Supplementary Fig S6. In Models 1 and 2, the odds of T2DM increased as the number of parities increased. A significant linear association between parity and T2DM was observed. Models 3 and 4 also exhibited significant linear associations between higher parity and higher odds for T2DM. Women with a clinical history of GDM had significantly higher odds of T2DM, compared to those without a clinical history of GDM. In Model 5, the association between parity and T2DM was attenuated compared to the results of Model 4, although a significant linear association remained. However, there was no significant difference between nulliparous women and women with parity=1.

3.4. Associations of parity with T2DM stratified by age

Considering the possibility that adjustment for age in a multiple logistic regression model could not remove the association between age and T2DM, we also performed the stratified analyses, stratifying women into age 50–64 years and age ≥65 years using Models 1–5.

The results of the association between parity and T2DM in women aged 50–64 years are shown in Supplementary Fig S7. Parity was not significantly associated with T2DM in Models 1 or 5. However, Models 2, 3 and 4 showed significant linear associations between higher parity and higher risk of T2DM. Women with a clinical history of GDM had significantly higher risk of T2DM in Models 3, 4, and 5.

The results of the association between parity and T2DM in women aged ≥65 years are shown in Supplementary Fig S8. Models 1, 2, 3, and 4 showed significant linear associations between higher parity and higher risk of T2DM. In Model 5, the association between parity and T2DM was weaker than

in Models 1, 2, 3, and 4. Women with a clinical history of GDM had significantly higher risk of T2DM in Models 3, 4, and 5.

In summary, the analyses stratified by age also showed that the association between parity and T2DM was weaker after adjusting for weight gain after 20 years of age.

4. References

1. Itabashi F, Hirata T, Kogure M, Narita A, Tsuchiya N, Nakamura T, et al. Combined associations of liver enzymes and obesity with diabetes mellitus prevalence: The Tohoku medical megabank community-based cohort study. J Epidemiol. 2022;32(5):2212–27. https://doi.org/10.2188/jea.JE20200384

2. Obesity: preventing and managing the global epidemic. Report of a WHO consultation. World Health Organ Tech Rep Ser. 2000;894:i-xii,1-253.

3. Nakaya N, Xie T, Scheerder B, Tsuchiya N, Narita A, Nakamura T, et al. Spousal similarities in cardiometabolic risk factors: A cross-sectional comparison between Dutch and Japanese data from two large biobank studies. Atherosclerosis. 2021;334:85–92. https://doi.org/10.1016/j.atherosclerosis.2021.08.03

4. Shozu M, Ishikawa H, Horikawa R, Sakakibara H, Izumi SI, Ohba T, et al. Nomenclature of primary amenorrhea: A proposal document of the Japan Society of Obstetrics and Gynecology committee for the redefinition of primary amenorrhea. J Obstet Gynaecol Res. 2017;43(11):1738–1742. https://doi.org/10.1111/jog.13442

5. Heianza Y, Kato K, Fujihara K, Tanaka S, Kodama S, Hanyu O , et al. Role of sleep duration as a risk factor for Type 2 diabetes among adults of different ages in Japan: the Niigata Wellness Study. Diabet Med. 2014;31(11):1363–1367. https://doi.org/10.1111/dme.12555

6. Nanri A, Mizoue T, Noda M, Goto A, Sawada N, Tsugane S. Menstrual and reproductive factors and type 2 diabetes risk: The Japan Public Health Center-based Prospective Study. J Diabetes Investig. 2019;10(1):147–153. https://doi.org/10.1111/jdi.12853

Supplementary Table S1. Differences in characteristics between women who were analyzed and excluded owing to missing data or clinically improbable data

Variables	Women who were analyzed (N=30,116)	Women who were excluded (N=10,493)	*P*-value
**Parity, N (%)**			<0.001
0	3,199 (10.6)	300 (2.9)	
1	3,185 (10.6)	893 (8.5)	
2	13,899 (46.2)	3,860 (36.8)	
3	8,345 (27.7)	2,333 (22.2)	
≥4	1,488 (4.9)	439 (4.2)	
Missing	0 (0.0)	2,668 (25.4)	
**Age, years**	59.0 (11.4)	60.5 (11.8)	<0.001
**Category of age, N (%)**			<0.001
20–29.9 years	431 (1.4)	161 (1.5)	
30–39.9 years	2,297 (7.6)	747 (7.1)	
40–49.9 years	3,217 (10.7)	939 (8.9)	
50–59.9 years	5,780 (19.2)	1,625 (15.5)	
60–69.9 years	13,578 (45.1)	4,413 (42.1)	
≥70 years	4,813 (16.0)	2,608 (24.9)	
**Waist circumference, cm**	81.2 (9.4)	82.5 (9.8)	<0.001
**Waist circumference ≥90 cm, N (%)**			<0.001
No	25,186 (83.6)	8,233 (78.5)	
Yes	4,930 (16.4)	2,144 (20.4)	

**Variables**	**Women who were analyzed (N=30,116)**	**Women who were excluded (N=10,493)**	***P*-value**
Missing	0 (0.0)	116 (1.1)	
**Height, cm**	153.2 (5.9)	152.3 (6.1)	<0.001
**Current body weight, kg**	53.7 (9.0)	54.6 (9.4)	<0.001
**Current BMI, kg/m^2**	22.9 (3.7)	23.5 (3.9)	<0.001
**Category of current BMI, N (%)**			<0.001
Underweight (<18.5 kg/m^2)	2,623 (8.7)	724 (6.9)	
Normal range (18.5–24.9 kg/m^2)	20,207 (67.1)	6,499 (61.9)	
Obese ≥25.0 kg/m^2)	7,286 (24.2)	3,244 (30.9)	
Missing	0 (0.0)	26 (0.2)	
**Body weight at 20 years, kg**	51.2 (7.5)	51.1 (7.4)	0.457
**BMI at 20 years, kg/m^2**	21.8 (3.1)	21.9 (3.2)	0.128
**Category of BMI at 20 years, N (%)**			<0.001
Underweight (<18.5 kg/m^2)	2,450 (8.1)	494 (4.7)	
Normal range (18.5–24.9 kg/m^2)	24,603 (81.7)	4,853 (46.2)	
Obese (≥25.0 kg/m^2)	3,063 (10.2)	696 (6.6)	
Missing	0 (0.0)	4,450 (42.4)	
**Body weight gain after 20 years, kg**	2.6 (8.9)	3.2 (9.2)	<0.001
**Physical activity level, METS, Median (IQR)**	27.8 (21.5–36.9)	27.1 (20.4–36.6)	0.007
**Smoking status, N (%)**			<0.001
Never smoker	25,041 (83.1)	7,179 (68.4)	
Ever smoker	2,599 (8.6)	729 (6.9)	

**Variables**	**Women who were analyzed (N=30,116)**	**Women who were excluded (N=10,493)**	***P*-value**
Current smoker	1,885 (6.3)	558 (5.3)	
Missing	591 (2.0)	2,027 (19.3)	
**Alcohol consumption, N (%)**			<0.001
Never drinker	18,879 (62.7)	5,810 (55.4)	
Ever drinking	517 (1.7)	140 (1.3)	
Current drinking	10,462 (34.7)	2,789 (26.6)	
Missing	258 (0.9)	1,754 (16.7)	
**T2DM prevalence, N (%)**	2,015 (6.7)	716 (6.8)	0.656
**Hypertension prevalence, N (%)**	10,526 (35.0)	3,875 (36.9)	<0.001
**Own birth weight, N (%)**			<0.001
<2,500 g	2,813 (9.3)	765 (7.3)	
2,500–3,499 g	12,694 (42.2)	3,021 (28.8)	
≥3,500 g	1,263 (4.2)	297 (2.8)	
Unknown	12,012 (39.9)	3,495 (33.3)	
Missing	1,334 (4.4)	2,915 (27.8)	
**History of thyroid dysfunction, N (%)**			<0.001
No	28,248 (93.8)	4,476 (42.7)	
Yes	1,541 (5.1)	384 (3.7)	
Missing	327 (1.1)	5,633 (53.7)	

Variables	Women who were analyzed (N=30,116)	Women who were excluded (N=10,493)	*P*-value
**History of endometriosis, N (%)**			<0.001
No	28,412 (94.3)	4,666 (44.5)	
Yes	1,414 (4.7)	316 (3.0)	
Missing	290 (1.0)	5,511 (52.5)	
**History of mental diseases, N (%)**			<0.001
No	28,704 (95.3)	4,522 (43.1)	
Yes	1,073 (3.6)	296 (2.8)	
Missing	339 (1.1)	5,675 (54.1)	
**Breastfeeding experience, N (%)**			<0.001
No	5,977 (19.8)	1,280 (12.2)	
Yes	23,898 (79.4)	5,803 (55.3)	
Missing	241 (0.8)	3,410 (32.5)	
**Use of oral contraceptives, N (%)**			<0.001
No	28,322 (94.0)	6,750 (64.3)	
Yes	914 (3.0)	215 (2.0)	
Missing	880 (2.9)	3,528 (33.6)	
**Use of hormone replacement therapy, N (%)**			<0.001
No	27,383 (90.9)	6,609 (63.0)	
Yes	1,967 (6.5)	415 (4.0)	
Missing	766 (2.5)	3,469 (33.1)	

Variables	Women who were analyzed (N=30,116)	Women who were excluded (N=10,493)	*P*-value
**≥15 years at menarche, N (%)**			<0.001
<15 years	24,315 (80.7)	5,303 (50.5)	
≥15 years	5,525 (18.3)	1,965 (18.7)	
Missing	276 (0.9)	3,225 (30.7)	
**≥35 years at last delivery, N (%)**			<0.001
<35 years	23,132 (76.8)	5,963 (56.8)	
≥35 years	3,256 (10.8)	655 (6.2)	
Missing	3,728 (12.4)	3,875 (36.9)	
**Menstrual cycle, N (%)**			<0.001
Regular	23,291 (77.3)	5,105 (48.7)	
Irregular	5,310 (17.6)	1,272 (12.1)	
Missing	1,515 (5.0)	4,116 (39.2)	
**History of GDM, N (%)**			<0.001
No	30,029 (99.7)	4,750 (45.3)	
Yes	87 (0.3)	4 (0.0)	
Missing	0 (0.0)	5,739 (54.7)	
**History of HDP, N (%)**			<0.001
No	28,798 (95.6)	4,669 (44.5)	
Yes	1,303 (4.3)	333 (3.2)	
Missing	15 (0.0)	5,491 (52.3)	

**Variables**	**Women who were analyzed (N=30,116)**	**Women who were excluded (N=10,493)**	***P*-value**
**Family history of type 2 DM, N (%)**			<0.001
No	26,448 (87.8)	4,611 (43.9)	
Yes	3,377 (11.2)	710 (6.8)	
Missing	291 (1.0)	5,172 (49.3)	
**Family history of hypertension, N (%)**			<0.001
No	18,487 (61.4)	4,395 (41.9)	
Yes	11,464 (38.1)	2,645 (25.2)	
Missing	165 (0.5)	3,453 (32.9)	
**Marital status, N (%)**			<0.001
Married	23,504 (78.0)	6,332 (60.3)	
Unmarried	1,887 (6.3)	363 (3.5)	
Divorced	1,373 (4.6)	398 (3.8)	
Widowed	3,183 (10.6)	1,114 (10.6)	
Missing	169 (0.6)	2,286 (21.8)	
**Highest level of education, N (%)**			<0.001
Low	5,482 (18.2)	2,565 (24.4)	
Medium	20,169 (67.0)	5,100 (48.6)	
High	4,162 (13.8)	735 (7.0)	
Missing	303 (1.0)	2,093 (19.9)	

Variables	Women who were analyzed (N=30,116)	Women who were excluded (N=10,493)	*P*-value
**Skipping breakfast, N (%)**			<0.001
Not skipping breakfast	26,962 (89.5)	7,610 (72.5)	
Skipping breakfast	2,691 (8.9)	760 (7.2)	
Missing	463 (1.5)	2,123 (20.2)	
**Average sleeping time/day, N (%)**			<0.001
<7 hours	22,387 (74.3)	6,239 (59.5)	
≥7 and <8 hours	5,917 (19.6)	1,786 (17.0)	
≥8 hours	1,783 (5.9)	585 (5.6)	
Missing	29 (0.1)	1,883 (17.9)	
**Nap time, N (%)**			<0.001
Not taking a nap	18,765 (62.3)	5,084 (48.5)	
Nap time <1 hour/day	9,049 (30.0)	2,759 (26.3)	
Nap time ≥1 hour/day	2,167 (7.2)	688 (6.6)	
Missing	135 (0.4)	1,962 (18.7)	
**Number of relocations after the GEJE, N (%)**			<0.001
0	22,767 (75.6)	5,566 (53.0)	
1	2,232 (7.4)	759 (7.2)	
2	1,498 (5.0)	630 (6.0)	
3	1,268 (4.2)	477 (4.5)	
≥4	717 (2.4)	250 (2.4)	
Missing	1,634 (5.4)	2,811 (26.8)	

Variables	Women who were analyzed (N=30,116)	Women who were excluded (N=10,493)	*P*-value
**Year, N (%)**			<0.001
2013	5,036 (16.7)	5,267 (50.2)	
2014	13,599 (45.2)	2,941 (28.0)	
2015	11,481 (38.1)	2,278 (21.7)	
**Prefecture, N (%)**			<0.001
Miyagi	16,595 (55.1)	7,576 (72.2)	
Iwate	13,521 (44.9)	2,917 (27.8)	
**Menopause status, N (%)**			<0.001
Premenopausal	6,588 (21.9)	1,347 (12.8)	
Premature menopause (age <40 years at menopause)	902 (3.0)	236 (2.2)	
Postmenopausal (age ≥40 years at menopause)	22,082 (73.3)	4,703 (44.8)	
Missing	544 (1.8)	4,207 (40.1)	
**Type of menopause, N (%)**			<0.001
Premenopausal	6,588 (21.9)	1,327 (12.6)	
Natural menopause	19,051 (63.3)	3,416 (32.6)	
Menopause due to surgery of uterus and/or ovary	3,424 (11.4)	711 (6.8)	
Other	757 (2.5)	153 (1.5)	
Missing	296 (1.0)	4,886 (46.6)	

Continuous variables are shown as mean (SD) or median (IQR). Categorical variables are shown as numbers (percentages).

Abbreviations: BMI, body mass index; DM, diabetes mellitus; GDM, gestational diabetes mellitus; GEJE, Great East Japan Earthquake;

HDP, hypertensive disorders of pregnancy; IQR, interquartile range; SD, standard deviation; T2DM, type 2 diabetes mellitus; WC, waist circumference.
