# Supplementary material for: Association of Parity with Type 2 Diabetes Mellitus in Japan
Source: Reprod Sci. 2024 Dec 11;32(2):366–81. doi: 10.1007/s43032-024-01752-z (PMC11825537; doi:10.1007/s43032-024-01752-z)
Supplement: Supplementary file 2 — Supplementary Fig S1. Association of parity with T2DM in premenopausal women (Models 6 and 7). Supplementary Fig S2. Association of parity with T2DM in postmenopausal women (Models 6 and 7) (PDF 128 KB) [file 43032_2024_1752_MOESM2_ESM.pdf]

Supplementary Figure S1

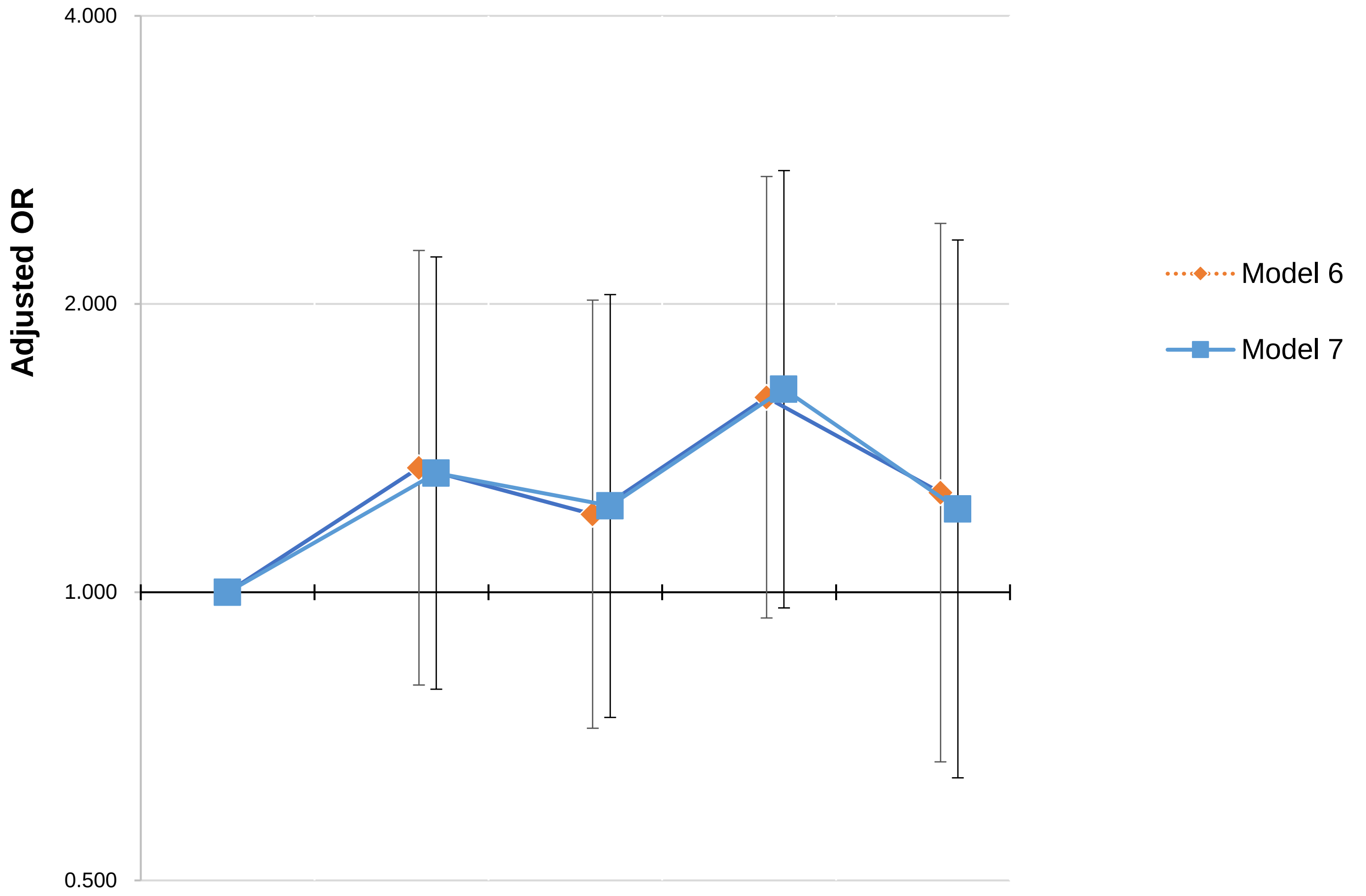

Model	Parity					*P*-value for trend	Clinical history of GDM	Current BMI, per 1-SD increase†	Waist circumference, per 1-SD increase†
	**0 (N=1,585)**	**1 (N=1,102)**	**2 (N=2,452)**	**3 (N=1,185)**	**≥4 (N=264)**				
**Cases of T2DM (%)**	36 (2.3)	21 (1.9)	39 (1.6)	37 (3.1)	7 (2.7)	0.36	NA	NA	NA
**Model 6** Adjusted OR (95% CI)	Reference	1.349 (0.800-2.275)	1.206 (0.721-2.019)	1.598 (0.940-2.718)	1.271 (0.665-2.428)	0.20	12.915 (5.324-31.334)	2.105 (1.866-2.376)	NA
**Model 7** Adjusted OR (95% CI)	Reference	1.332 (0.792-2.240)	1.231 (0.740-2.046)	1.630 (0.963-2.757)	1.222 (0.640-2.333)	0.20	12.534 (5.243-29.966)	NA	2.417 (2.098-2.785)

Supplementary Figure S1. Association of parity with T2DM in premenopausal women (Models 6 and 7)
†1-SD values were 3.0 kg/m^2 for current BMI and 9.8 cm for waist circumference.
Model 6: Adjusting for age, height, physical activity, marital status, smoking status, alcohol consumption, own birth weight, highest educational level, family history of T2DM, family history of hypertension, breastfeeding experience, use of oral contraceptives, use of hormone replacement therapy, thyroid dysfunction, endometriosis, mental disease, menstrual cycle, age at menarche (<15 years or ≥15 years), age at last delivery (<35 years or ≥35 years), sleeping time, nap time, year of study participation, Prefecture (Miyagi or Iwate), number of relocations after the GEJE, clinical history of GDM, and current BMI , as per 1-SD increase.
Model 7: Adjusting for age, height, physical activity, marital status, smoking status, alcohol consumption, own birth weight, highest educational level, family history of T2DM, family history of hypertension, breastfeeding experience, use of oral contraceptives, use of hormone replacement therapy, thyroid dysfunction, endometriosis, mental disease, menstrual cycle, age at menarche (<15 years or ≥15 years), age at last delivery (<35 years or ≥35 years), sleeping time, nap time, year of study participation, Prefecture (Miyagi or Iwate), number of relocations after the GEJE, clinical history of GDM, and waist circumference, as per 1-SD increase.
Abbreviations: BMI, body mass index; GEJE, Great East Japan Earthquake; CI, confidence interval; GDM, gestational diabetes mellitus; OR, odds ratio; NA, not applicable; T2DM, type 2 diabetes mellitus.

Supplementary Figure S2

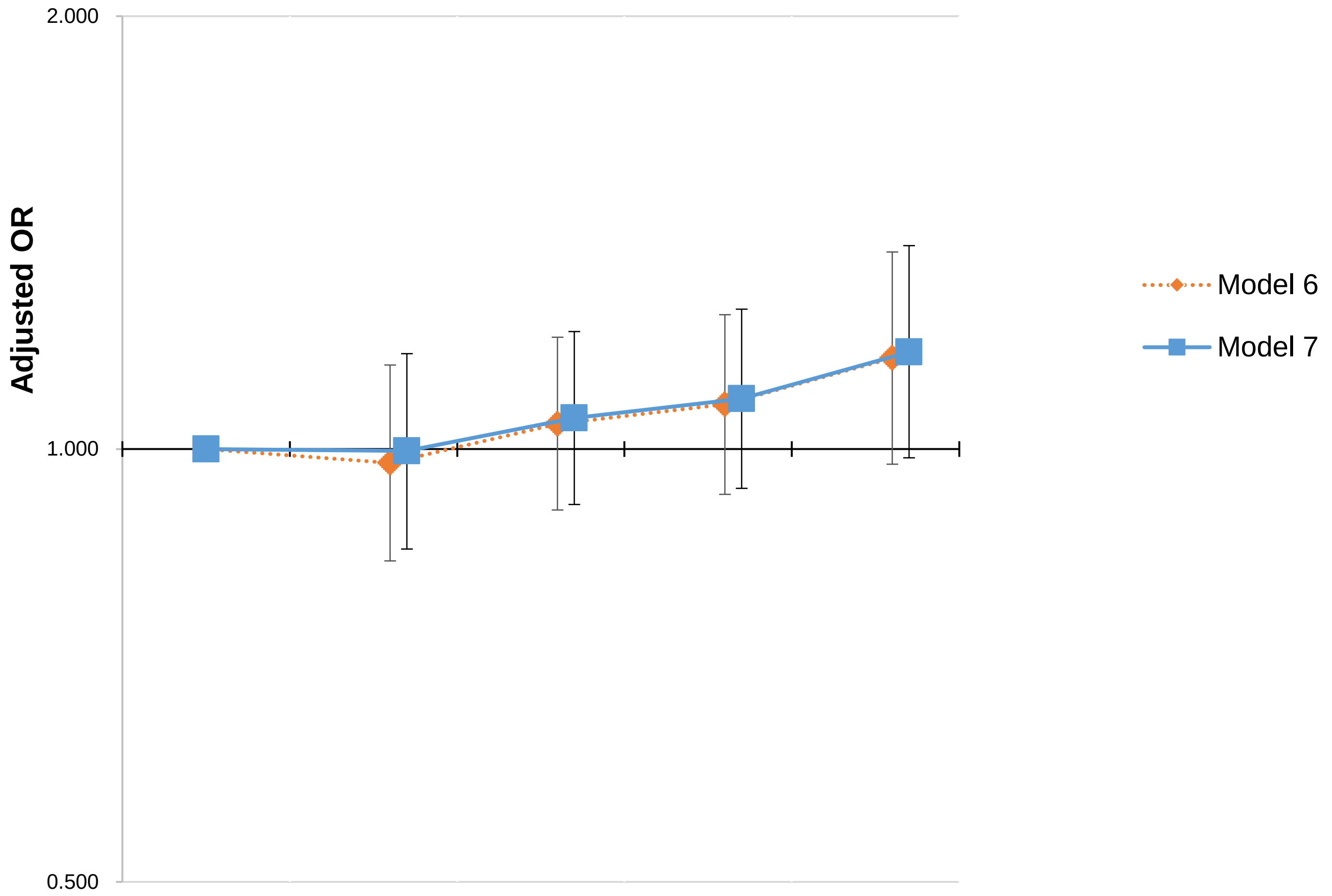

Model	Parity					*P*-value for trend	Clinical history of GDM	Current BMI, per 1-SD increase†	Waist circumference, per 1-SD increase†
	**0 (N=1,614)**	**1 (N=2,083)**	**2 (N=11,447)**	**3 (N=7,160)**	**≥4 (N=1,224)**				
**Cases of T2DM (%)**	108 (6.7)	150 (7.2)	877 (7.7)	612 (8.5)	128 (10.5)	<0.0001	NA	NA	NA
**Model 6** Adjusted OR (95% CI)	Reference	0.978 (0.836-1.144)	1.041 (0.907-1.196)	1.074 (0.930-1.240)	1.157 (0.976-1.371)	0.012	8.008 (3.679-17.432)	1.773 (1.695-1.854)	NA
**Model 7** Adjusted OR (95% CI)	Reference	0.997 (0.852-1.165)	1.051 (0.915-1.207)	1.084 (0.939-1.251)	1.168 (0.986-1.385)	0.013	8.055 (3.730-17.395)	NA	1.812 (1.728-1.901)

Supplementary Figure S2. Association of parity with T2DM in postmenopausal women (Models 6 and 7)

†1-SD values were 3.0 kg/m^2 for current BMI and 9.1 cm for waist circumference.

Model 6: Adjusting for age, height, physical activity, marital status, smoking status, alcohol consumption, own birth weight, highest educational level, family history of T2DM, family history of hypertension, breastfeeding experience, use of oral contraceptives, use of hormone replacement therapy, thyroid dysfunction, endometriosis, mental disease, menstrual cycle, age at menarche (<15 years or ≥15 years), age at last delivery (<35 years or ≥35 years), menopause age (<40 years or ≥40 years), sleeping time, nap time, year of study participation, Prefecture (Miyagi or Iwate), number of relocations after the GEJE, clinical history of GDM, and current BMI , as per 1-SD increase.

Model 7: Adjusting for age, height, physical activity, marital status, smoking status, alcohol consumption, own birth weight, highest educational level, family history of T2DM, family history of hypertension, breastfeeding experience, use of oral contraceptives, use of hormone replacement therapy, thyroid dysfunction, endometriosis, mental disease, menstrual cycle, age at menarche (<15 years or ≥15 years), age at last delivery (<35 years or ≥35 years), menopause age (<40 years or ≥40 years), sleeping time, nap time, year of study participation, Prefecture (Miyagi or Iwate), number of relocations after the GEJE, clinical history of GDM, and waist circumference, as per 1-SD increase .

Abbreviations: BMI, body mass index; CI, confidence interval; DM, diabetes mellitus; GDM, gestational diabetes mellitus; OR, odds ratio; SD, standard deviation; T2DM, type 2 diabetes mellitus; NA, not applicable.
