# Supplementary material for: Association of Parity with Type 2 Diabetes Mellitus in Japan
Source: Reprod Sci. 2024 Dec 11;32(2):366–81. doi: 10.1007/s43032-024-01752-z (PMC11825537; doi:10.1007/s43032-024-01752-z)
Supplement: Supplementary file 3 — Supplementary Fig S3. Combined analysis for investigation of interaction between a clinical history of GDM and parity in premenopausal women. Supplementary Fig S4. Combined analysis for investigation of interaction between a clinical history of GDM and parity in postmenopausal women (PDF 127 KB) [file 43032_2024_1752_MOESM3_ESM.pdf]

# Supplementary Figure S3

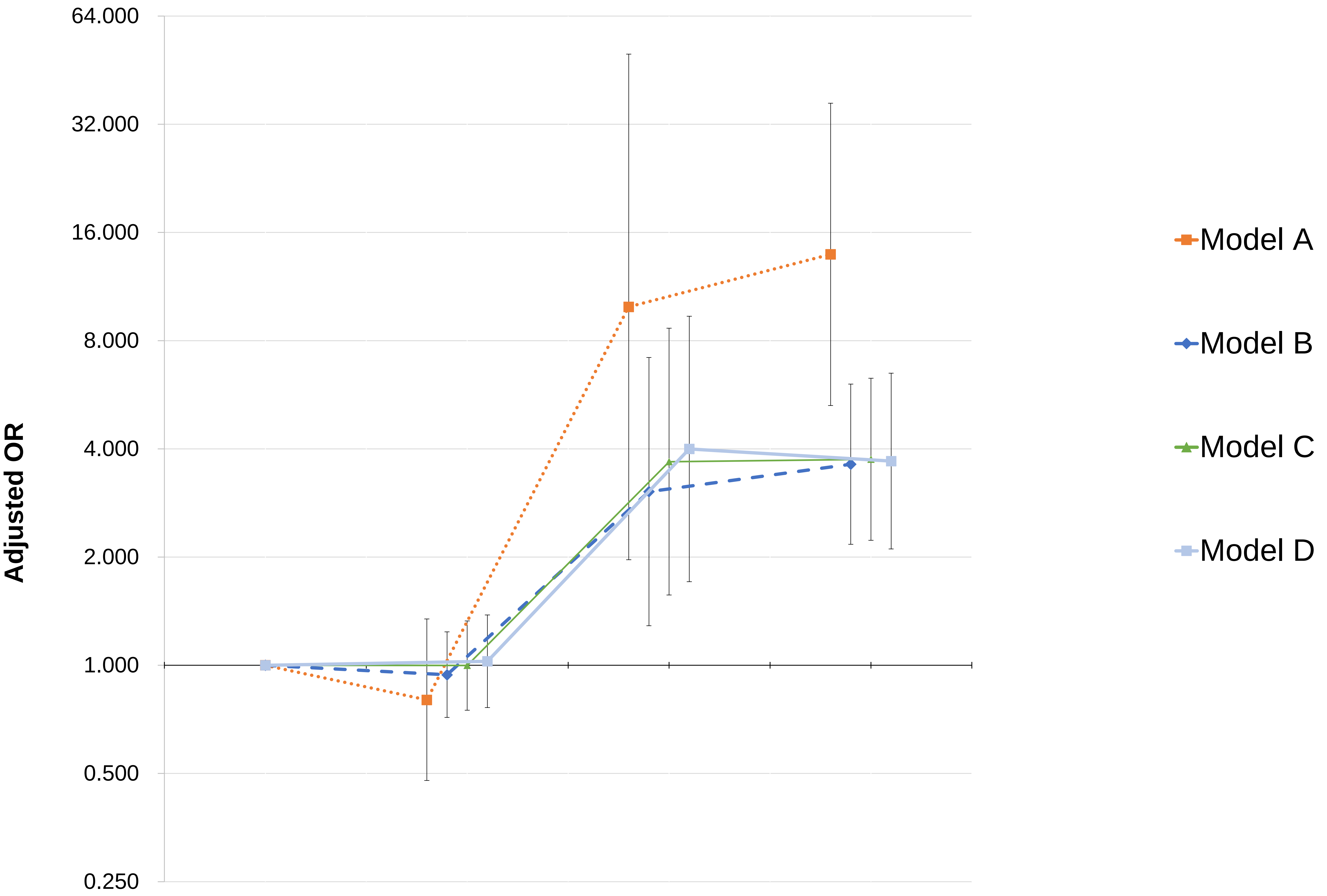


| Clinical history of GDM | No | | Yes | | *P*-value for interaction | BMI at 20-years-old, per 1-SD increase† | Weight gain after 20 years of age, per 1 kg increase |
|---|---|---|---|---|---|---|---|
| Parity | 1 (N=1,088) | ≥2 (N=3,861) | 1 (N=14) | ≥2 (N=40) | | | |
| Cases of T2DM (%) | 19 (1.75) | 76 (1.97) | 2 (14.29) | 7 (17.50) | NA | NA | NA |
| **Model A** Adjusted OR (95% CI) | Reference | 0.801 (0.478-1.345) | 9.931 (1.966-50.167) | 13.908 (5.282-36.621) | 0.63 | NA | NA |
| **Model B** Adjusted OR (95% CI) | Reference | 0.941 (0.716-1.239) | 3.041 (1.288-7.180) | 3.625 (2.170-6.056) | 0.96 | NA | NA |
| **Model C** Adjusted OR (95% CI) | Reference | 0.999 (0.750-1.329) | 3.685 (1.568-8.662) | 3.742 (2.226-6.289) | 0.97 | 1.664 (1.418-1.953) | NA |
| **Model D** Adjusted OR (95% CI) | Reference | 1.026 (0.763-1.380) | 3.998 (1.708-9.357) | 3.698 (2.107-6.493) | 0.84 | 2.321 (1.954-2.758) | 1.085 (1.064-1.106) |

**Supplementary Figure S3. Combined analysis for investigation of interaction between a clinical history of GDM and parity in premenopausal women**

†1-SD value was 2.8 kg/m$^2$ for BMI at 20-years-old.

Model A: Adjusting for age.

Model B: Model A plus covariates as follows: height, physical activity, marital status, smoking status, alcohol consumption, own birth weight, highest educational level, family history of T2DM, family history of hypertension, breastfeeding experience, use of oral contraceptives, use of hormone replacement therapy, thyroid dysfunction, endometriosis, mental disease, menstrual cycle, age at menarche (<15 years or ≥15 years), age at last delivery (<35 years or ≥35 years), sleeping time, nap time, year of study participation, Prefecture (Miyagi or Iwate), and number of relocations after the GEJE.

Model C: Model B plus BMI at 20-years-old, as per 1-SD increase.

Model D: Model C plus weight gain after 20 years of age, as per 1 kg increase.

Abbreviations: BMI, body mass index; CI, confidence interval; DM, diabetes mellitus; GDM, gestational diabetes mellitus; GEJE, Great East Japan Earthquake; OR, odds ratio; SD, standard deviation; T2DM, type 2 diabetes mellitus; NA, not applicable.

# Supplementary Figure S4

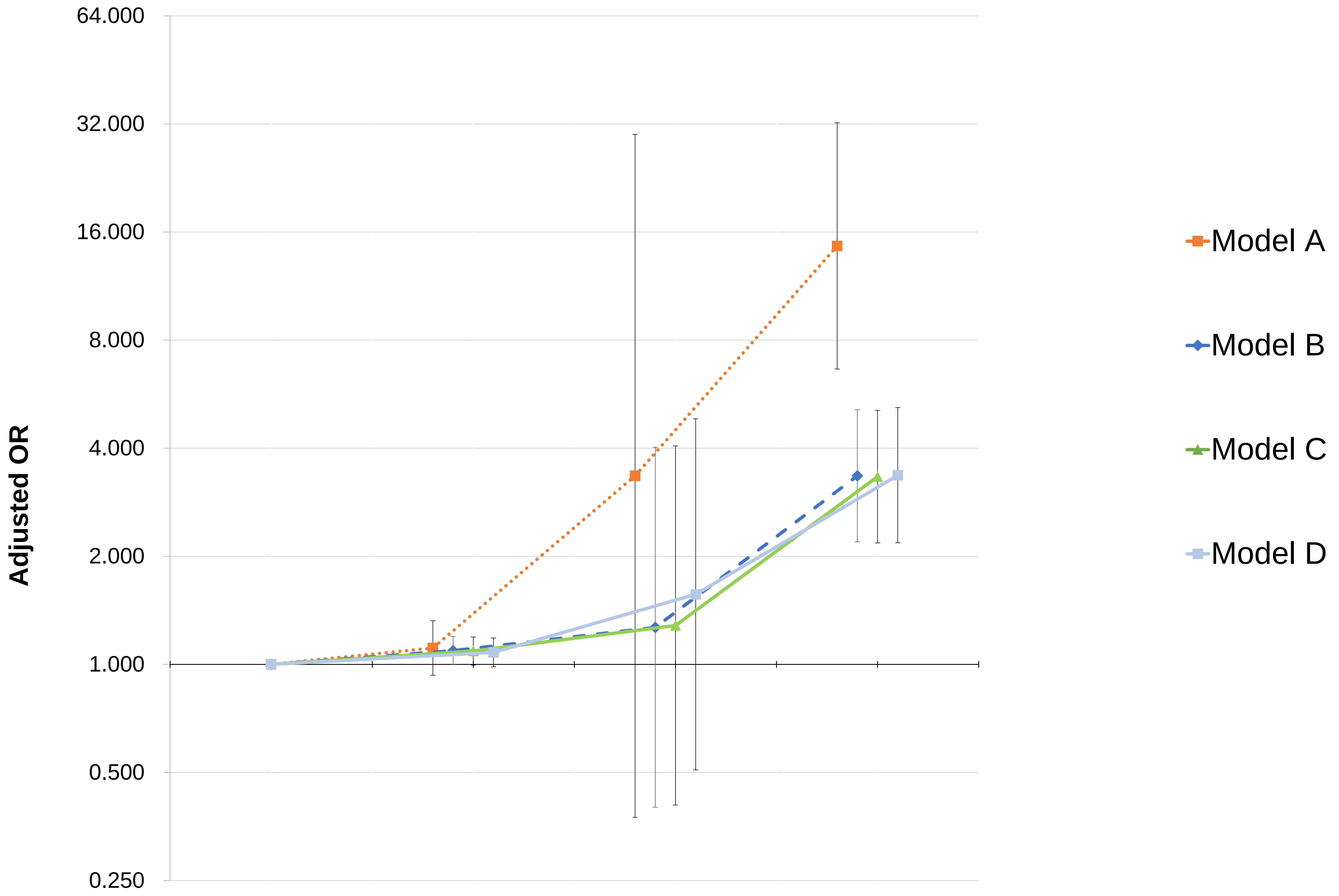


| Clinical history of GDM | No | | Yes | | *P*-value for interaction | BMI at 20-years-old, per 1-SD increase† | Weight gain after 20 years of age, per 1 kg increase |
|---|---|---|---|---|---|---|---|
| **Parity** | **1 (N=2,077)** | **≥2 (N=19,804)** | **1 (N=6)** | **≥2 (N=27)** | | | |
| **Cases of T2DM (%)** | 149 (7.17) | 1,604 (8.10) | 1 (16.67) | 13 (48.15) | NA | NA | NA |
| **Model A** Adjusted OR (95% CI) | Reference | 1.111 (0.933-1.323) | 3.351 (0.375-29.924) | 14.632 (6.644-32.227) | 0.15 | NA | NA |
| **Model B** Adjusted OR (95% CI) | Reference | 1.092 (0.998-1.195) | 1.268 (0.400-4.023) | 3.351 (2.194-5.120) | 0.26 | NA | NA |
| **Model C** Adjusted OR (95% CI) | Reference | 1.090 (0.996-1.192) | 1.283 (0.406-4.059) | 3.333 (2.179-5.097) | 0.16 | 1.097 (1.053-1.143) | NA |
| **Model D** Adjusted OR (95% CI) | Reference | 1.080 (0.985-1.185) | 1.567 (0.508-4.829) | 3.363 (2.180-5.189) | 0.25 | 1.569 (1.479-1.665) | 1.075 (1.069-1.082) |

**Supplementary Figure S4. Combined analysis for investigation of interaction between a clinical history of GDM and parity in postmenopausal women**

†1-SD value was 3.1 kg/$m^2$ for BMI at 20-years-old.

Model A: Adjusting for age.

Model B: Model A plus covariates as follows: height, physical activity, marital status, smoking status, alcohol consumption, own birth weight, highest educational level, family history of type 2 DM, family history of hypertension, breastfeeding experience, use of oral contraceptives, use of hormone replacement therapy, thyroid dysfunction, endometriosis, mental disease, menstrual cycle, age at menarche (<15 years or ≥15 years), age at last delivery (<35 years or ≥35 years), menopause age (<40 years or ≥40 years), sleeping time, nap time, year of study participation, Prefecture (Miyagi or Iwate), and number of relocations after the GEJE.

Model C: Model B plus BMI at 20-years-old, as per 1-SD increase.

Model D: Model C plus weight gain after 20 years of age, as per 1 kg increase.

Abbreviations: BMI, body mass index; CI, confidence interval; DM, diabetes mellitus; GDM, gestational diabetes mellitus; GEJE, Great East Japan Earthquake; OR, odds ratio; SD, standard deviation; T2DM, type 2 diabetes mellitus; NA, not applicable.
