# Supplementary material for: Association of Parity with Type 2 Diabetes Mellitus in Japan
Source: Reprod Sci. 2024 Dec 11;32(2):366–81. doi: 10.1007/s43032-024-01752-z (PMC11825537; doi:10.1007/s43032-024-01752-z)
Supplement: Supplementary file 5 — Supplementary Fig S7. Association of parity with T2DM in women aged 50 to 64. Supplementary Fig S8. Association of parity with T2DM in women aged 65 or more (PDF 141 KB) [file 43032_2024_1752_MOESM5_ESM.pdf]

Supplementary Figure S7

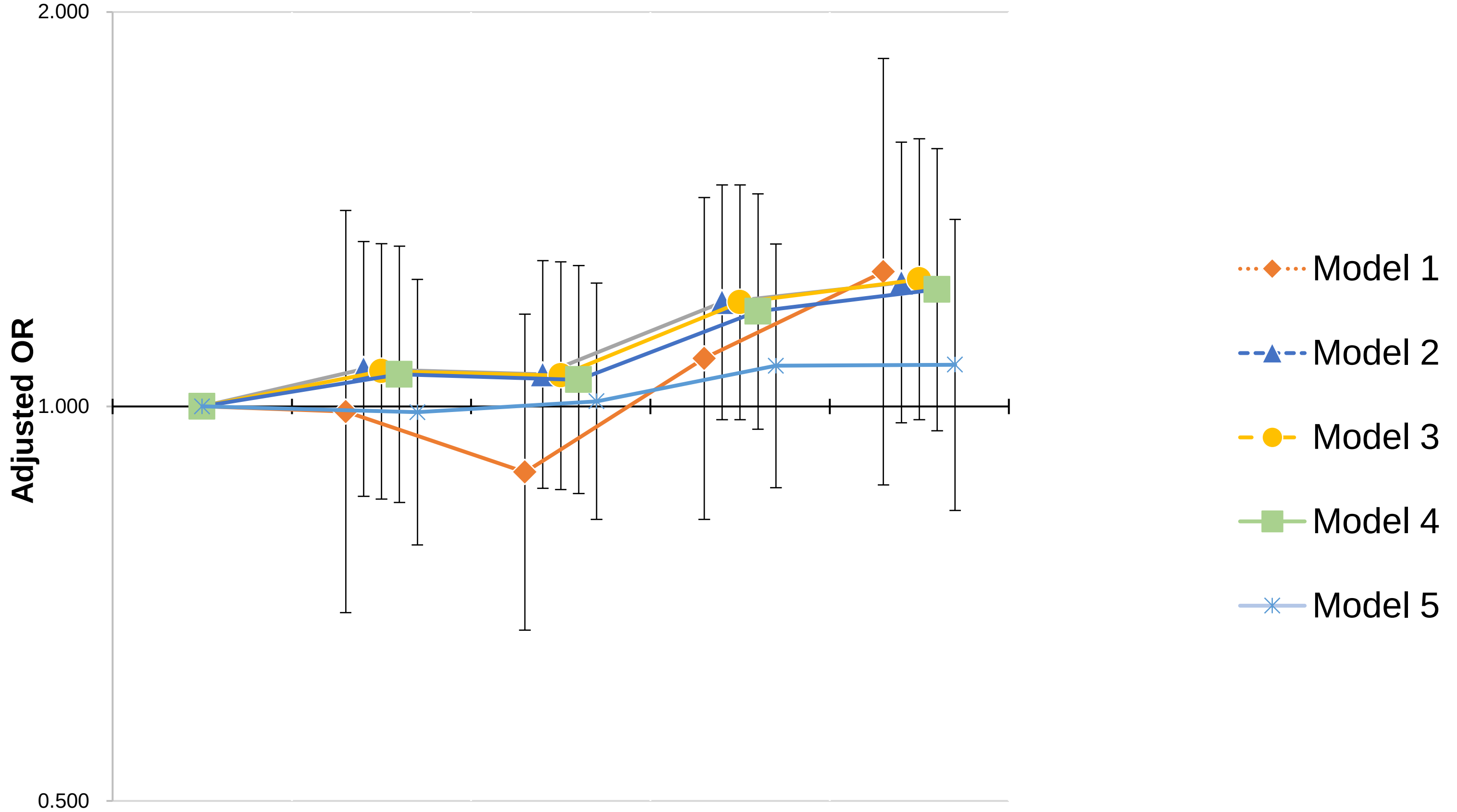

Model	Parity					Clinical history of GDM	BMI at 20-years-old, per 1-SD increase†	Weight gain after 20 years of age, per 1 kg increase	P-value for trend
	0 (N=1,063)	1 (N=1,091)	2 (N=5,677)	3 (N=3,930)	≥4 (N=699)				
Cases of T2DM (%)	64 (6.0)	68 (6.2)	333 (5.9)	276 (7.0)	55 (7.9)	NA	NA	NA	0.037
**Model 1** Adjusted OR (95% CI)	Reference	0.991 (0.696-1.411)	0.891 (0.675-1.176)	1.088 (0.820-1.443)	1.267 (0.871-1.843)	NA	NA	NA	0.087
**Model 2** Adjusted OR (95% CI)	Reference	1.068 (0.854-1.336)	1.058 (0.866-1.292)	1.201 (0.977-1.476)	1.243 (0.972-1.591)	NA	NA	NA	0.003
**Model 3** Adjusted OR (95% CI)	Reference	1.064 (0.850-1.331)	1.056 (0.864-1.289)	1.201 (0.977-1.476)	1.250 (0.977-1.600)	7.531 (2.960-19.163)	NA	NA	0.002
**Model 4** Adjusted OR (95% CI)	Reference	1.058 (0.845-1.325)	1.048 (0.858-1.281)	1.182 (0.961-1.453)	1.228 (0.958-1.573)	7.362 (2.858-18.963)	1.287 (1.196-1.385)	NA	0.005
**Model 5** Adjusted OR (95% CI)	Reference	0.990 (0.784-1.250)	1.009 (0.820-1.242)	1.074 (0.867-1.330)	1.076 (0.833-1.389)	6.381 (2.411-16.890)	1.936 (1.765-2.124)	1.090 (1.081-1.099)	0.17

Supplementary Figure S7. Association of parity with T2DM in women aged 50 to 64
†1-SD value was 2.7 kg/m^2 for BMI at 20-years-old.
Model 1: Adjusting for age.
Model 2: Model 1 plus covariates as follows: height, physical activity, marital status, smoking status, alcohol consumption, own birth weight, highest educational level, family history of T2DM, family history of hypertension, breastfeeding experience, use of oral contraceptives, use of hormone replacement therapy, thyroid dysfunction, endometriosis, mental disease, menstrual cycle, age at menarche (<15 years or ≥15 years), age at last delivery (<35 years or ≥35 years), sleeping time, nap time, year of study participation, Prefecture (Miyagi or Iwate), and number of relocations after the GEJE.
Model 3: Model 2 plus a clinical history of GDM.
Model 4: Model 3 plus BMI at 20-years-old, as per 1-SD increase.
Model 5: Model 4 plus weight gain after 20 years of age, as per 1 kg increase.
Abbreviations: BMI, body mass index; CI, confidence interval; DM, diabetes mellitus; GDM, gestational diabetes mellitus; GEJE, Great East Japan Earthquake; OR, odds ratio; SD, standard deviation; T2DM, type 2 diabetes mellitus; NA, not applicable.

Supplementary Figure S8

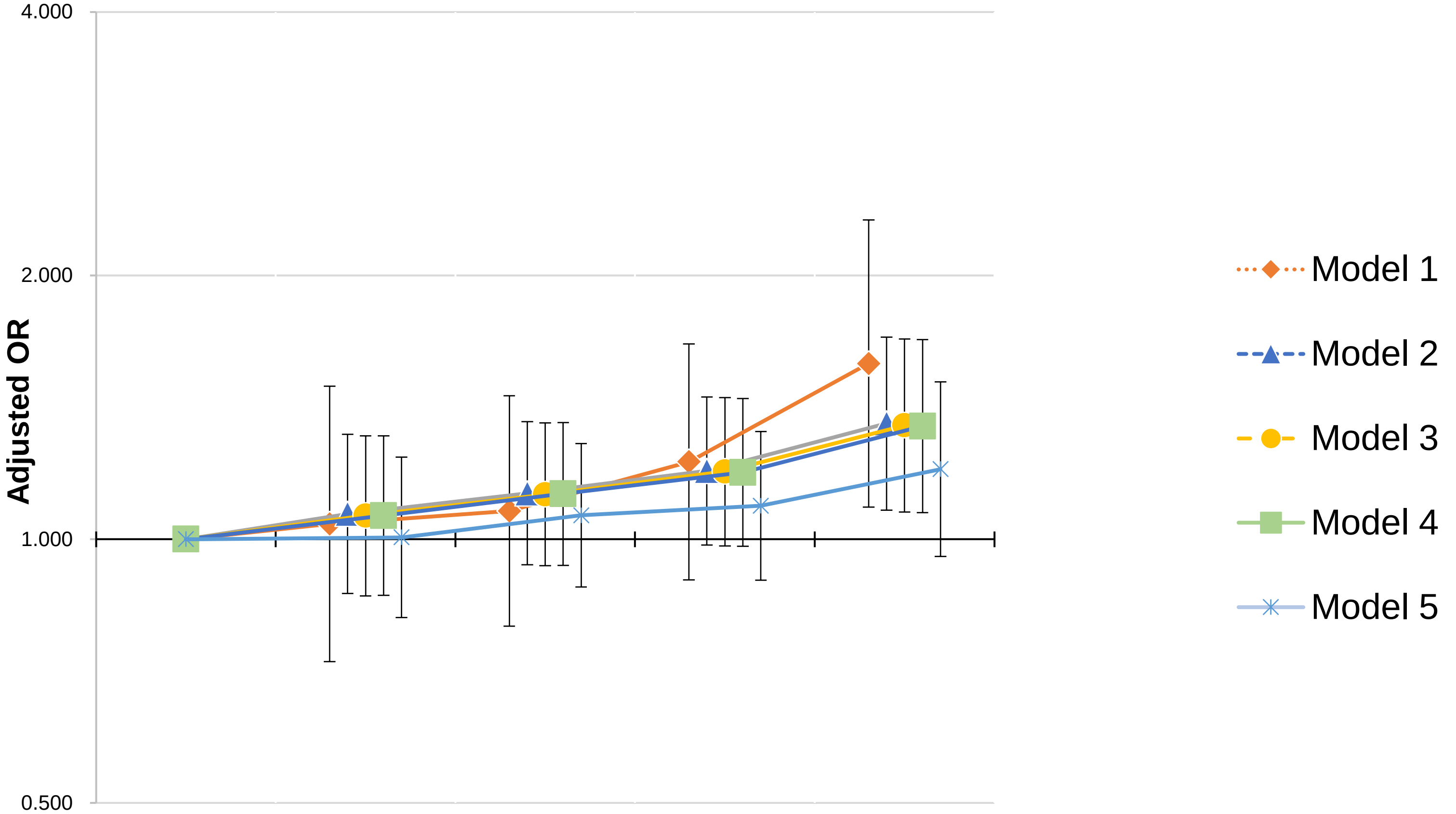

Model	Parity					Clinical history of GDM	BMI at 20-years-old, per 1-SD increase†	Weight gain after 20 years of age, per 1 kg increase	P-value for trend
	0 (N=595)	1 (N=1,017)	2 (N=6,080)	3 (N=3,436)	≥4 (N=582)				
**Cases of T2DM (%)**	50 (8.4)	90 (8.9)	552 (9.1)	350 (10.2)	75 (12.9)	NA	NA	NA	0.003
**Model 1** Adjusted OR (95% CI)	Reference	1.041 (0.725-1.495)	1.077 (0.796-1.458)	1.226 (0.899-1.671)	1.587 (1.088-2.316)	NA	NA	NA	0.003
**Model 2** Adjusted OR (95% CI)	Reference	1.069 (0.867-1.318)	1.128 (0.935-1.362)	1.197 (0.985-1.454)	1.356 (1.080-1.701)	NA	NA	NA	0.001
**Model 3** Adjusted OR (95% CI)	Reference	1.064 (0.862-1.312)	1.125 (0.933-1.358)	1.195 (0.983-1.451)	1.349 (1.074-1.693)	7.733 (2.052-29.148)	NA	NA	0.001
**Model 4** Adjusted OR (95% CI)	Reference	1.064 (0.863-1.313)	1.127 (0.934-1.359)	1.192 (0.982-1.448)	1.346 (1.073-1.690)	7.785 (2.063-29.370)	1.041 (0.982-1.103)	NA	0.001
**Model 5** Adjusted OR (95% CI)	Reference	1.005 (0.814-1.241)	1.065 (0.882-1.286)	1.092 (0.898-1.328)	1.202 (0.956-1.512)	11.266 (3.043-41.713)	1.462 (1.345-1.589)	1.063 (1.054-1.071)	0.045

Supplementary Figure S8. Association of parity with T2DM in women aged 65 or more
†1-SD value was 3.4 kg/m^2 for BMI at 20-years-old.
Model 1: Adjusting for age.
Model 2: Model 1 plus covariates as follows: height, physical activity, marital status, smoking status, alcohol consumption, own birth weight, highest educational level, family history of T2DM, family history of hypertension, breastfeeding experience, use of oral contraceptives, use of hormone replacement therapy, thyroid dysfunction, endometriosis, mental disease, menstrual cycle, age at menarche (<15 years or ≥15 years), age at last delivery (<35 years or ≥35 years), sleeping time, nap time, year of study participation, Prefecture (Miyagi or Iwate), and number of relocations after the GEJE.
Model 3: Model 2 plus a clinical history of GDM.
Model 4: Model 3 plus BMI at 20-years-old, as per 1-SD increase.
Model 5: Model 4 plus weight gain after 20 years of age, as per 1 kg increase.
Abbreviations: BMI, body mass index; CI, confidence interval; DM, diabetes mellitus; GDM, gestational diabetes mellitus; GEJE, Great East Japan Earthquake; OR, odds ratio; SD, standard deviation; T2DM, type 2 diabetes mellitus; NA, not applicable.
